# Interleukins 4 and 13 and Their Receptors Are Differently Expressed in Gastrointestinal Tract Cancers, Depending on the Anatomical Site and Disease Advancement, and Improve Colon Cancer Cell Viability and Motility

**DOI:** 10.3390/cancers12061463

**Published:** 2020-06-04

**Authors:** Iwona Bednarz-Misa, Dorota Diakowska, Izabela Szczuka, Paulina Fortuna, Agnieszka Kubiak, Joanna Rosińczuk, Małgorzata Krzystek-Korpacka

**Affiliations:** 1 Department of Medical Biochemistry, Wroclaw Medical University, 50-368 Wroclaw, Poland; iwona.bednarz-misa@umed.wroc.pl (I.B.-M.); izabela.szczuka@umed.wroc.pl (I.S.); paulina.fortuna@umed.wroc.pl (P.F.); a.kubiak@umed.wroc.pl (A.K.); 2 Department of Gastrointestinal and General Surgery, Wroclaw Medical University, 50-368 Wroclaw, Poland; dorota.diakowska@umed.wroc.pl; 3 Department of Nervous System Diseases, Wroclaw Medical University, 51-618 Wroclaw, Poland; joanna.rosinczuk@umed.wroc.pl

**Keywords:** colorectal cancer, esophageal squamous cell carcinoma, gastric adenocarcinoma, immunotherapy, molecular margin, interleukin

## Abstract

Immunosuppressive interleukins (IL)-4 and 13 may directly promote cancer but neither their status nor role in gastrointestinal tract is clarified. We aim at quantifying ILs and their receptors in paired normal-tumor samples (*n* = 49/51) and sera (*n* = 263), using immunoassays and RTqPCR, and screening for their effect on colonic cancer cells. Both ILs were elevated locally at protein level in all cancers but only *IL13* transcripts in colon were upregulated. Interleukin and their receptor expression reflected cancer pathology to varying degrees, with the association frequently inverse and manifested in non-cancerous tissue. Positive correlation with cancer-promoting genes *BCL2*, *BCLxL*, *HIF1A*, *VEGFA*, *ACTA2*, *CCL2*, *PTGS2*, and *CDKN1A*, but not *Ki67*, was demonstrated, particularly for ILs’ receptors. Circulating IL-4 was elevated in all, while IL-13 only in colorectal or esophageal cancers, reflecting their advancement. *IL4Ra* and *IL13Ra1* transcripts were downregulated by hypoxia and, in Caco-2, also by IL-4. Interleukin stimulation slightly improved colonic cancer cell viability, weakly upregulating *BCL2* and *Ki67* in HCT116 and HT-29. It affected cell motility more markedly and was consistently accompanied by upregulation of claudin-2. Gastrointestinal tract cancers are associated with IL-4 and IL-13 upregulation, which may facilitate cancer growth. Targeting both interleukins as an antineoplastic strategy warrants further investigation.

## 1. Introduction

Cancers of the gastrointestinal tract (GIT), including esophageal squamous cell carcinoma (ESCC), gastric adenocarcinoma (GC), and colorectal adenocarcinoma (CRC), are among the leading causes of death worldwide [1]. CRC alone accounted for an estimated 1,850,000 new cancer cases in 2018 and was the second leading cause of cancer-related deaths [1]. Tumor resection, combined with chemotherapy or radiotherapy, remains the main treatment option but may fail to improve the outcome in patients with metastatic or resistant cancers. Harnessing the immune system to fight cancer is seen as an attractive alternative to chemotherapy and radiation as a more precise approach, potentially better tolerated and yielding durable remission [2,3]. However, immunotherapies seem to be less effective than might be expected from preclinical studies, to which a highly immunosuppressive tumor microenvironment significantly contributes [4].

The ability to escape immunosurveillance is considered an emerging hallmark of cancer [5]. Interleukins (IL)-4 and IL-13 are among the immunomodulatory cytokines believed to play a prominent role in tumor immunity [4]. They might be secreted by tumor-supporting cells and aid tumor growth by inducing and activating immunosuppressive phenotypes in surrounding immune cells [4,6]. Moreover, a growing body of evidence shows that, if relevant receptors are expressed, they might promote tumor growth also directly, by inducing cancer cell proliferation and migration and/or by protecting transformed cells from apoptosis. Immune cells express receptors consisting of IL-4Rα and common γc subunits (type I IL-4 receptor). The receptor on epithelial cancer cells, referred to as type II IL-4 receptor, consist of IL-4Rα and IL-13Rα1. IL-13 may interact with type II IL-4R or bind to its specific IL-13Rα2 receptor [7,8]. However, IL-13Rα2 has been considered a decoy receptor unable to transduce signal because of the lack of significant cytoplasmic domain [9]. Still, others have proven its relevance for cancer invasion and metastasis [10,11].

The expression of both interleukins and their receptors in various tumors is increasingly being demonstrated (reviewed in [7,8]). Moreover, possible autocrine mode of action has been investigated [12]. However, available literature data concerning IL-4 and IL-13 in CRC and upper GIT cancers are fragmentary and frequently inconsistent. Contradicting findings have been reported, especially concerning the interleukins’ status at local and systemic level and their association with the disease advancement [13,14,15]. Consequently, both tumor promoting [10,16,17,18,19,20,21,22] and protective roles [14,15,23,24,25,26,27,28,29,30] have been advocated for IL-4 and IL-13. In the light of existing controversies, the aim of present study is a comprehensive comparative analysis of IL-4 and IL-13 expression at mRNA and protein level, local and systemic, in CRC as compared to esophageal and gastric cancers. Interleukin quantification was accompanied by transcriptional analysis of expression patterns of type II IL-4 receptor subunits (IL-4Rα and IL-13Rα1). In addition, clinical samples were screened for potential associations with representative indicators of: angiogenesis (hypoxia-inducible factor 1α (*HIF1A*) and vascular endothelial growth factor A (*VEGFA*)); apoptosis (*BCL2*, *BCLxL*, and p21^CIP1/WAF1^ (*CDKN1A*)); proliferation (*Ki67*); inflammation (*CCL2*, cyclooxygenase-2 (*PTGS2*)*,* and inducible nitric oxide synthase (*NOS2*)); metabolic reprogramming (glucose transporter (*GLUT1*), ornithine decarboxylase from nitric oxide/polyamine pathway (*ODC1*), and *NOS2*) and epithelial-mesenchymal transition (EMT) (smooth muscle α-2 actin (*ACTA2*)) as well as with tight junction proteins (zonula occludens-1 (*TJP1*) and claudin-2 *(CLDN2*)). The possibility of cause-effect relationship in CRC as well as the interleukin effect on cell viability and motility is then explored in vitro.

## 2. Results

### 2.1. Local Expression of Interleukins and Their Receptors

IL-4 and IL-13 protein expression was determined in tissue homogenates using dedicated immunoassays and expressed per gram of tissue, while IL4/IL13 and IL4Ra/IL13Ra1 transcripts were quantified using reversely transcribed real time (quantitative) PCR methodology and SYBR green chemistry, as described in detail in Materials and Methods section.

#### 2.1.1. Characteristics of Study Population

Patients’-matched samples of tumor and tumor-adjacent macroscopically normal tissue were collected intraoperatively from patients with CRC, ESCC, or GC for mRNA (*n* = 51) and protein analysis (*n* = 49). Patients in each cancer group were matched regarding age, sex distribution, and the disease advancement (Table 1 and Table 2).

#### 2.1.2. Concentration of IL-4 Protein and Expression of *IL4* and *IL4Ra* Transcripts in CRC as Compared to Upper GIT Cancers

**Figure 1:**
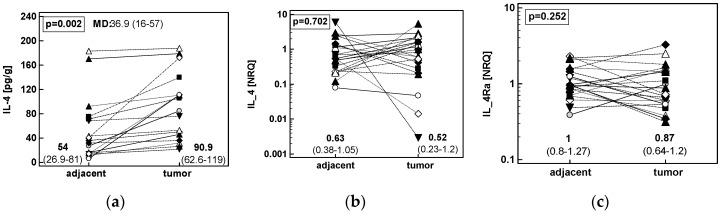
Patients’-matched analysis of tumor and tumor-adjacent tissue expression of: (**a**) IL-4 protein in CRC (*n* = 17); (**b**) *IL4* mRNA in CRC (*n* = 21); (**c**) *IL4Ra* mRNA in CRC (*n* = 21). Data were analyzed as logs using *t*-test for paired samples and presented as geometric means with 95% confidence interval (*CI*). Mean difference in protein concentration (MD) between groups is accompanied by 95% *CI*. Transcriptomic data are presented as normalized relative quantities (NRQ). CRC, colorectal cancer; ESCC, esophageal squamous cell carcinoma; GC, gastric adenocarcinoma.

Paired comparison of IL-4 in adjacent and tumor colonic tissue showed significantly higher protein concentration in tumors but similar expression level of *IL4* and *IL4Ra* transcripts (Figure 1).

**Figure 2:**
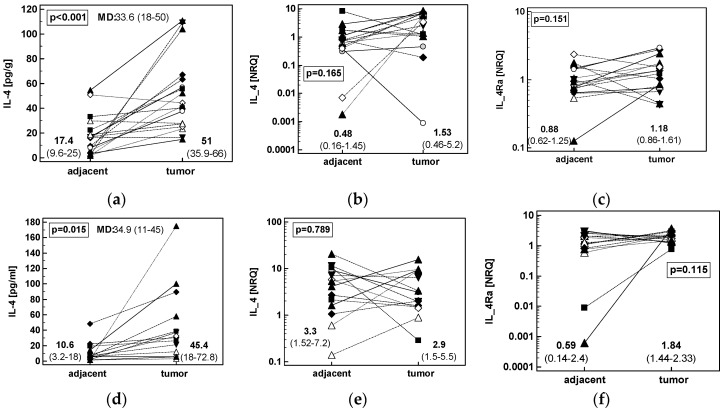
Patients’-matched analysis of tumor and tumor-adjacent tissue expression of: (**a**) IL-4 protein in ESCC (*n* = 18); (**b**) *IL4* mRNA in ESCC (*n* = 16); (**c**) *IL4Ra* mRNA in ESCC (*n* = 16); (**d**) IL-4 protein in GC (*n* = 14); (**e**) *IL4* mRNA in GC (*n* = 14); (**f**) *IL4Ra* mRNA in GC (*n* = 14). Data were analyzed as logs using *t*-test for paired samples and presented as geometric means with 95% confidence interval (*CI*). Mean difference in protein concentration (MD) between groups is accompanied by 95% *CI*. Transcriptomic data are presented as normalized relative quantities (NRQ). CRC, colorectal cancer; ESCC, esophageal squamous cell carcinoma; GC, gastric adenocarcinoma.

Similar to CRC, patients with GC or ESCC had significantly higher IL-4 protein concentration in tumors. The differences in *IL4* and *IL4Ra* transcripts between adjacent and tumor tissue were non-significant (Figure 2).

**Figure 3:**
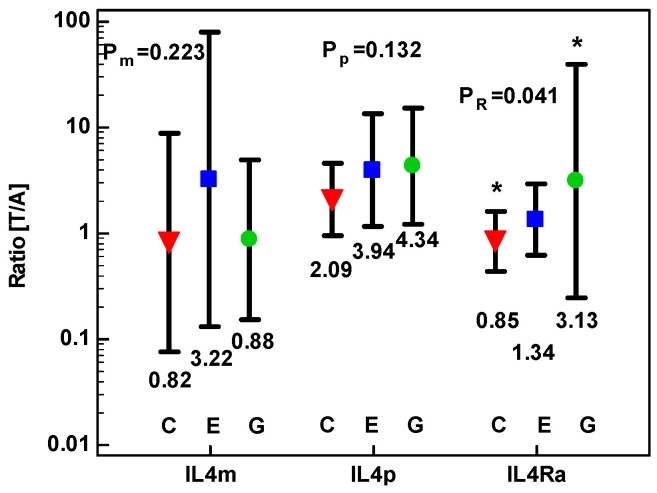
Effect of anatomical site on fold-change in *IL4* mRNA (IL4m), IL-4 protein (IL4p), and *IL4Ra* expression in tumor as compared to adjacent tissue [T/A]. Data were analyzed as logs using one-way ANOVA and presented as geometric means with 95% confidence interval (whiskers). Red triangles represent mean values in colorectal cancers (denoted as C); blue squares represent mean values in esophageal squamous cell carcinoma (denoted as E); green circles represent mean values in gastric adenocarcinoma (denoted as G). *p* values for *IL4* mRNA analysis are denoted as P_m_, for IL-4 protein analysis as P_p_, and for *IL4Ra* mRNA analysis as P_R_. Statistically significant between-group differences are marked with asterisks (*).

The level of IL-4 protein upregulation in tumor tissue was comparable between anatomical sites, whether expressed as mean difference (MD) or fold-change (tumor-to-adjacent ratio) (Figure 3). Fold-change in *IL4* transcripts was comparable in CRC and upper GIT cancers as well. In turn, *IL4Ra* was more markedly upregulated in tumors from GC than CRC patients, despite high dispersion of values around mean in GC (Figure 3).

**Figure 4:**
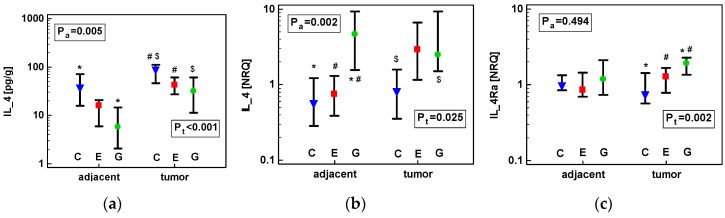
Effect of anatomical site on tumor and tumor-adjacent tissue expression of: (**a**) IL-4 protein; (**b**) *IL4* mRNA; (**c**) *IL4Ra* mRNA. Data analyzed as logs using one-way ANOVA and presented as geometric means with 95% confidence interval (whiskers). Blue triangles represent mean values in colorectal cancers (denoted as C); red squares represent mean values in esophageal squamous cell carcinoma (denoted as E); green circles represent mean values in gastric adenocarcinoma (denoted as G). *p* values for the analysis in adjacent tissue are denoted as P_a_ and for tumor tissue as P_t_. Statistically significant differences between groups are marked with symbols of the same type (*, #, etc.). Transcriptomic data presented as normalized relative quantities (NRQ).

However, there were significant differences between cancer types in IL-4 protein and *IL4* and *IL4Ra* transcript numbers, in both tumor and adjacent tissue, when they were analyzed directly and not as a fold-change. The absolute IL-4 protein concentration in adjacent tissue was significantly higher in colonic than gastric tissue. In tumors, it was higher in colonic as compared to both gastric and esophageal tumors (Figure 4a). Contrary to IL-4 protein, *IL4* mRNA expression in non-cancerous tissue was the highest in GC. It was also higher in GC as compared to CRC tumors (Figure 4b). The expression of *IL4Ra* mRNA differed significantly between anatomical sites only for tumor tissue, with *IL4Ra* expression significantly higher in GC as compared to CRC and ESCC tumors (Figure 4c).

Fold-change in IL-4 protein concentration in CRC was not related to any evaluated pathological data. In ESCC, fold-increase in IL-4 protein concentration in tumor was strongly directly correlated with tumor grade. In GC, it was significantly higher in M1 cancers and tended to correlate positively with cancer stage (Table 3).

Interestingly, positive correlation between tumor grade and fold-change in IL-4 protein concentration in ESCC patients resulted from a negative correlation observed in adjacent non-cancerous tissue (ρ = −0.60, *p* = 0.009) and not from an increasing IL-4 accumulation in tumor. Similarly, there was an inverse relationship between tumor grade and IL-4 protein concentration in non-cancerous tissue in CRC (ρ = -0.55, *p* = 0.021). Those results might imply decreasing number of IL-4 secreting cells in tumor surrounding tissue with increasing cancer aggressiveness.

Fold-change in *IL4* transcript number was significantly lower in ESCC patients with lymph node metastasis. In addition, it tended to be inversely related with tumor extension in CRC (Table 4). In GC, *IL4* transcript number in tumor tissue tended to positively correlate with TNM stage (ρ = 0.48, *p* = 0.082) and to be higher in tumors from patients with lymph node metastasis (4.5 vs. 1.6, *p* = 0.091).

There was no significant association between fold change in expression level of *IL4Ra* and pathological data in any cancer type. Only *IL4Ra* fold-change tended to be lower in ESCC patients with lymph node metastasis (Table 5).

In turn, there was an inverse correlation between IL-4 protein concentration and expression of *IL4* transcripts in tumor (r = −0.40, *p* = 0.019) and tumor-adjacent tissue (r = −0.43, *p* = 0.011) as well as between their fold-change (tumor-to-adjacent; r = −0.42, *p* = 0.015).

#### 2.1.3. Concentration of IL-13 Protein and Expression of *IL13* and *IL13Ra1* Transcripts in CRC as Compared to upper GIT Cancers

**Figure 5:**
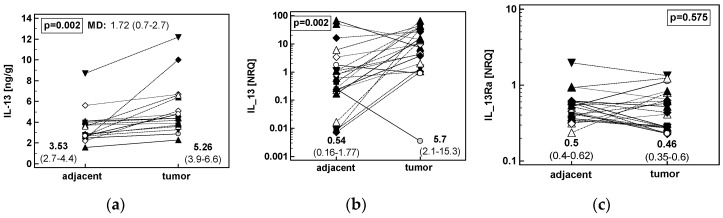
Patients’-matched analysis of tumor and tumor-adjacent tissue expression of: (**a**) IL-13 protein in CRC (*n* = 17); (**b**) *IL13* mRNA in CRC (*n* = 21); (**c**) *IL13Ra1* mRNA in CRC (*n* = 21). Data were analyzed as logs using *t*-test for paired samples and presented as geometric means with 95% confidence interval (*CI*). Mean difference in protein concentration (MD) between groups is accompanied by 95% *CI*. Transcriptomic data are presented as normalized relative quantities (NRQ). CRC, colorectal cancer; ESCC, esophageal squamous cell carcinoma; GC, gastric adenocarcinoma.

Paired comparison of IL-13 in adjacent and tumor colonic tissue showed significantly higher protein concentration and *IL13* transcript number in tumors but similar expression level of *IL13Ra1* transcripts (Figure 5).

**Figure 6:**
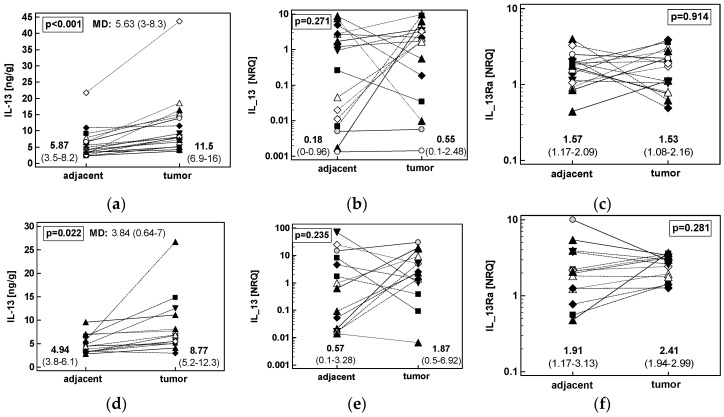
Patients’-matched analysis of tumor and tumor-adjacent tissue expression of: (**a**) IL-13 protein in ESCC (*n* = 18); (**b**) *IL13* mRNA in ESCC (*n* = 16); (**c**) *IL13Ra1* mRNA in ESCC (*n* = 16); (**d**) IL-13 protein in GC (*n* = 14); (**e**) *IL13* mRNA in GC (*n* = 14); (**f**) *IL13Ra1* mRNA in GC (*n* = 14). Data were analyzed as logs using *t*-test for paired samples and presented as geometric means with 95% confidence interval (*CI*). Mean difference in protein concentration (MD) between groups is accompanied by 95% *CI*. Transcriptomic data are presented as normalized relative quantities (NRQ). CRC, colorectal cancer; ESCC, esophageal squamous cell carcinoma; GC, gastric adenocarcinoma.

Similarly, patients with GC or ESCC had significantly higher IL-13 protein concentration in tumors but the mean difference in its concentration was lower in CRC than upper GIT cancers (Figure 6). The differences in both *IL13* and *IL13Ra1* transcripts between adjacent and tumor tissue were non-significant (Figure 6).

**Figure 7:**
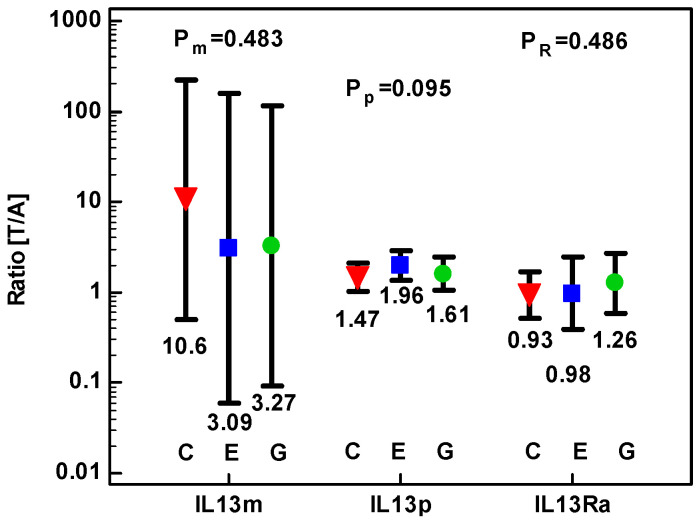
Effect of anatomical site on tumor-to-adjacent expression ratios of *IL13* mRNA (IL13m), IL-13 protein (IL13p), and *IL13Ra1*. Data analyzed as logs using one-way ANOVA and presented as geometric means with 95% confidence interval (whiskers). Red triangles represent mean values in colorectal cancers (denoted as C); blue squares represent mean values in esophageal squamous cell carcinoma (denoted as E); green circles represent mean values in gastric adenocarcinoma (denoted as G). *p* values for *IL13* mRNA analysis are denoted as P_m_, for IL-13 protein analysis as P_p_, and for *IL13Ra1* mRNA analysis as P_R_.

Fold-change in IL-13 protein concentration and expression of *IL13* and *IL13Ra1* transcripts was comparable in CRC and upper GIT cancers (Figure 7).

**Figure 8:**
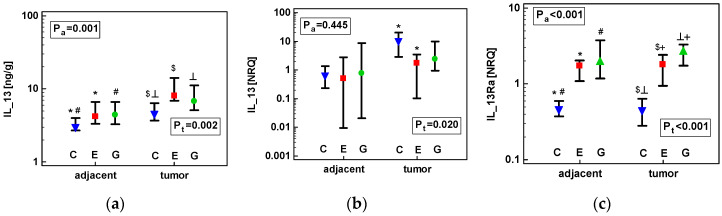
Effect of anatomical site on tumor and tumor-adjacent tissue expression of: (**a**) IL-13 protein; (**b**) *IL13* mRNA; (**c**) *IL13Ra1* mRNA. Data analyzed as logs using one-way ANOVA and presented as geometric means with 95% confidence interval (whiskers). Blue triangles represent mean values in colorectal cancers (denoted as C); red squares represent mean values in esophageal squamous cell carcinoma (denoted as E); green circles represent mean values in gastric adenocarcinoma (denoted as G). *p* values for the analysis in adjacent tissue are denoted as P_a_ and for tumor tissue as P_t_. Statistically significant differences between groups are marked with symbols of the same type (*, #, etc.). Transcriptomic data presented as normalized relative quantities (NRQ).

However, there were significant anatomical site-associated differences in IL-13 protein concentrations (Figure 8a) and *IL13Ra1* expression (Figure 8c). In both adjacent and tumor tissue (Figure 8), they were the lowest in CRC patients. In addition, tumor tissue from ESCC patients had significantly lower *IL13Ra1* (Figure 8c) and *IL13* expression (Figure 8b) as compared to GC patients.

Fold-change in IL-13 protein concentration positively correlated with TNM stage in CRC patients and tended to be higher in N1 patients (Table 6). The association with TNM in CRC resulted from IL-13 decrease along with increasing TNM in adjacent tissue (ρ = −0.42, *p* = 0.093) rather than its increase in tumor (ρ = 0.20, *p* = 0.436). No significant association between fold-change in IL-13 protein concentration and cancer pathology was observed in ESCC and GC (Table 6). However, the interleukin concentration changed with TNM and/or T in both cancers. Yet, as the change occurred in both tumor and non-cancerous tissue, it was not reflected by a fold-change. In ESCC, IL-13 protein concentration increased along with advancing TNM in tumor (ρ = 0.56, *p* = 0.016) and tumor-adjacent tissue (ρ = 0.52, *p* = 0.026) and tended to positively correlate with T in tumors (ρ = 0.43, *p* = 0.078). In GC, it was negatively correlated with T, significantly so in adjacent tissue (ρ = −0.62, *p* = 0.019) and non-significantly in tumors (ρ = −0.45, *p* = 0.104). It also tended to correlate with TNM (ρ = −0.50, *p* = 0.072 in adjacent and ρ = −0.50, *p* = 0.069 in tumor tissue). In addition, IL-13 protein concentration was significantly lower in GC patients with lymph node metastasis when evaluated either in tumor (5.5 vs. 11.4, *p* = 0.008) or non-cancerous adjacent tissue (3.9 vs. 5.8, *p* = 0.039).

Fold-change in *IL13* expression was significantly lower in CRC patients with lymph node metastasis and inversely related to the TNM stage (Table 7). The inverse association with lymph node involvement was a result of higher *IL13* transcript expression in tumor-adjacent non-cancerous tissue in N1 than N0 CRC patients (3 vs. 0.2, *p* = 0.028) and a tendency toward its lower expression in tumors from N1 patients (2.2 vs. 9.2, *p* = 0.158). Similarly, *IL13* expression in non-cancerous adjacent tissue tended to correlate positively with TNM (ρ = 0.42, *p* = 0.059).

There were no significant associations between *IL13Ra1* expression and pathology in any of the examined cancers (Table 8). Likewise, receptor expression did not correlate with pathological findings in tumor and adjacent tissues.

There was no correlation between IL-13 protein concentration and *IL13* transcript number in tumor or adjacent tissue and neither was the fold-change correlated. Similarly, there was no significant correlation between transcript numbers of *IL13* and *IL13Ra1* or between IL-13 protein concentration and *IL13Ra1*.

#### 2.1.4. Correlation Pattern between *IL4*, *IL4Ra*, *IL13*, and *IL13Ra1* and the Expression of Mediators Relevant for Cancer Growth in CRC Patients

Transcriptional analysis of correlation patterns was conducted on clinical samples from CRC patients to reveal possible association between expression level of *IL4*, *IL4Ra*, *IL13*, and *IL13Ra1* and that of representative mediators/markers associated with cancer development and progression. We investigated mediators of angiogenesis (hypoxia inducible factor 1α (*HIF1A*) and vascular endothelial growth factor A (*VEGFA*)), apoptosis (antiapoptotic *BCL2* and *BCLxL* and cell cycle regulator p21^CIP1/WAF1^ (*CDKN1A*)), proliferation (*Ki67*), inflammation (monocyte chemoattractant protein 1 (*CCL2*), cyclooxygenase-2 (*PTGS2*) and inducible nitric oxide synthase (*NOS2*)), metabolic reprogramming (glucose transporter 1(*GLUT1*), ornithine decarboxylase (*ODC1*) and *NOS2*), EMT (smooth muscle α-2 actin (*ACTA2*)), and tight junction proteins (zonula occludens-1 (*TJP1*) and claudin-2 (*CLDN2*)).

Fold-change in expression ratio of *IL13Ra1* positively correlated with fold-change in *CDKN1A* and *VEGFA* and tended to correlate with *HIF1A*, while *IL13* displayed no significant correlations. Fold-change in expression ratio of *IL4Ra* positively correlated with fold-change in *ACTA2*, *CCL2*, *CDKN1A*, *PTGS2*, *TJP1*, and *VEGFA* and tended to positively correlate with *HIF1A* but was inversely related to *CLDN2* and *Ki67*. Fold-change in *IL4* tended to be positively correlated with fold-change in *ACTA2* (Table 9).

The correlation analysis of gene expression in tumor tissue showed *IL13Ra1* to correlate positively with *ACTA2*, *CCL2*, *CDKN1A*, *HIF1A*, *TJP1*, and *VEGFA* and tended to correlate with *BCL2* while *IL13* tended to correlate positively with *PTGS2* and *VEGFA*. Expression of *IL4Ra* in CRC tumors correlated positively with *ACTA2*, *BCL2*, *CCL2*, *CDKN1A*, *HIF1A*, *TJP1*, and *VEGFA* and tended to correlate positively with *BCLxL* and negatively with *CLDN2* and *NOS2,* while *IL4* tended to correlate negatively with *NOS2* (Table 10).

The correlation analysis of gene expression in non-cancerous tumor-adjacent tissue showed fewer correlations. *IL13Ra1* expression correlated positively with that of *TJP1* and tended to correlate with *NOS2* and *VEGFA* while *IL13* expression correlated positively with *PTGS2* and tended to correlate negatively with *BCL2*, *GLUT1*, and *NOS2*. *IL4Ra* expression correlated positively with *ACTA2* and *PTGS2* and tended to correlate positively with *TJP1* but was correlated negatively with *Ki67*. *IL4* expression correlated positively with *CCL2* and tended to correlate positively with *HIF1A* and *PTGS2* (Table 10).

### 2.2. Systemic Concentrations of IL-4 and IL-13 in CRC and upper GIT Cancers

Systemic concentrations of IL-4 and IL-13 were measured in serum samples using flow cytometry-based Luminex xMAP^®^ technology. Serum samples were collected from 227 cancer patients upon hospital admission prior any treatment and from 36 apparently healthy controls. Data on demography and pathology are summarized in Table 11.

**Figure 9:**
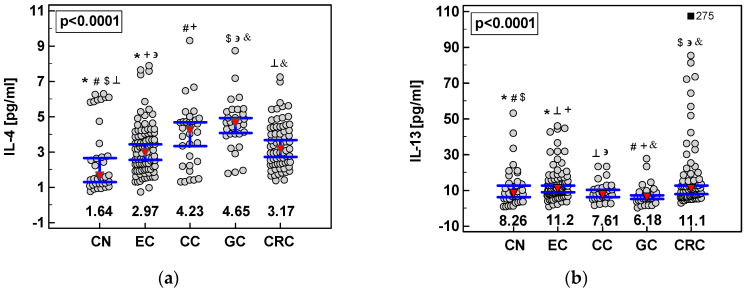
Systemic concentration of interleukins in gastrointestinal tract cancers: (**a**) IL-4; (**b**) IL-13. Data presented as medians (red triangles) with 95% confidence interval (whiskers) and analyzed using Kruskal-Wallis *H* test. Statistically significant differences between groups are marked with symbols of the same type (*, #, etc.). CN, healthy controls; EC, patients with esophageal squamous cell carcinoma; CC, patients with adenocarcinoma of gastric cardia; GC, patients with gastric adenocarcinoma; CRC, patients with colorectal cancer. An outlying observation is marked by a closed square.

As compared to controls, IL-4 was significantly elevated in all cancers. Patients with adenocarcinomas of gastric cardia (CC) and GC patients had significantly higher IL-4 concentration than ESCC patients and GC patients—significantly higher as compared to CRC patients as well (Figure 9a). There was no correlation between IL-4 and the disease advancement or tumor grade in any of examined cancers.

As compared to controls, IL-13 concentration was significantly higher in ESCC and CRC but lower in GC patients. The ESCC and CRC patients had significantly higher IL-13 concentration also as compared to CC and GC patients (Figure 9b). Systemic IL-13 concentration was significantly higher in ESCC patients with lymph node involvement (11.6 pg/mL (9.8–13.7) in N ≥ 1 vs. 6.57 pg/mL (3.9–11.1) in N0, *p* = 0.014) and tended to be higher in ESCC patients with distant metastases (11.8 pg/mL (9.4-15) in M1 vs. 8.03 pg/mL (5.7–11.2) in M0, *p* = 0.089). It positively correlated with tumor extension (T; ρ = 0.26, *p* = 0.014) and tended to correlate with ESCC stage (TNM; ρ = 0.19, *p* = 0.075). In CRC, systemic IL-13 significantly and positively correlated with tumor grade (ρ = 0.27, *p* = 0.039) and tended to correlate with tumor extension (ρ = 0.21, *p* = 0.080) and CRC stage (ρ = 0.21, *p* = 0.077).

### 2.3. Effect of Exogenous IL-4 and IL-13 on Colon Cancer Cells—Preliminary Findings

#### 2.3.1. Expression of *IL4* and *IL13* and Their Receptors (*IL4Ra* and *IL13Ra1*) in Colon Cancer Cell Lines: Effect of Nutritional Stress, Chemically Induced Hypoxia, and IL-4 and IL-13

Three colonic cancer cell lines cultured in our laboratory, namely Caco-2, HCT 116, and HT-29 as well as biobanked cDNA from LoVo, SW480, and SW620 cells were screened for the expression of *IL4*, *IL13*, *IL4Ra*, and *IL13Ra1*. *IL4* transcripts were expressed at low level in Caco-2 but were only barely detectable in HCT 116, HT-29, LoVo, and SW620 and undetectable in SW480 cells. *IL13* transcripts were expressed at equally low level in Caco-2 and were undetectable in other cell lines.

**Figure 10:**
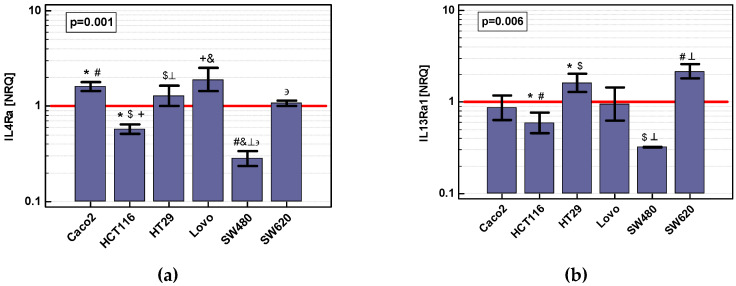
Relative expression of *IL4Ra* and *IL13Ra1* in colon cancer cells lines: (**a**) *IL4Ra*; (**b**) *IL13Ra1*. Data analyzed as logs using one-way ANOVA and presented as geometric means ± SE (*n* = 2–6) of normalized relative quantities [NRQ]. Expression levels were normalized against *GAPDH* expression and presented in relation to geometric mean of gene expression across all samples (represented by horizontal red line). Statistically significant differences between groups are marked with symbols of the same type (*, #, etc.).

In turn, all cell lines expressed *IL4Ra* and *IL13Ra1*. *IL4Ra* expression was significantly higher in Caco-2, HT-29, and LoVo cells than in HCT 116 and SW480. In addition, *IL4Ra* expression in SW620 was higher than in SW480 cells. *IL13Ra1* expression was significantly higher in HT-29 and SW620 than in HCT 116 and SW480 cells (Figure 10).

Subsequently, potential effects of nutritional stress (serum withdrawal) and chemically induced hypoxia on *IL4Ra* and *IL13Ra1* expression in Caco-2, HCT 116, and HT-29 cells was evaluated.

**Figure 11:**
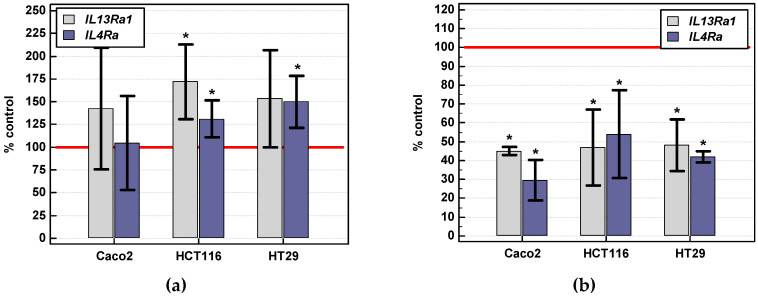
*IL13Ra1* and *IL4Ra* expression under stress conditions: (**a**) serum withdrawal; (**b**) chemically induced hypoxia. Bars represent mean (*n* = 2–4) of relative expression of receptor transcripts in cells grown with 0.5% FBS or stimulated with 200 µM CoCl_2_ for 24 h as compared to controls (cells grown with 10% FBS or unstimulated), expressed as a percentage. Reference expression level of control/untreated cells (100%) is represented by red horizontal line. Statistically significant differences in treated cells as compared to their respective controls are marked with asterisks (*) (analyzed using *t*-test for one mean).

*IL4Ra* expression was slightly upregulated by serum starvation in HCT 116 and HT-29, but not Caco-2 cells. *IL13Ra1* expression was significantly upregulated by serum starvation solely in HCT 116 cells (Figure 11a).

Chemically induced hypoxia (200 µM CoCl_2_) reduced *IL4Ra* expression to 30% in Caco-2 cells and to 54% in HCT 116 and 42 in HT29 cells. *IL13Ra1* expression dropped to 45% in Caco-2 and to 47% in HCT 116 and 48% in HT-29 cells (Figure 11b).

IL-4 has been shown to stimulate the expression of IL-4Ra in immune cells [31]. Therefore, the potential effect of IL-4 stimulation on *IL4Ra* and *IL13Ra1* expression and of IL-13 on *IL13Ra1* expression was evaluated.

**Figure 12:**
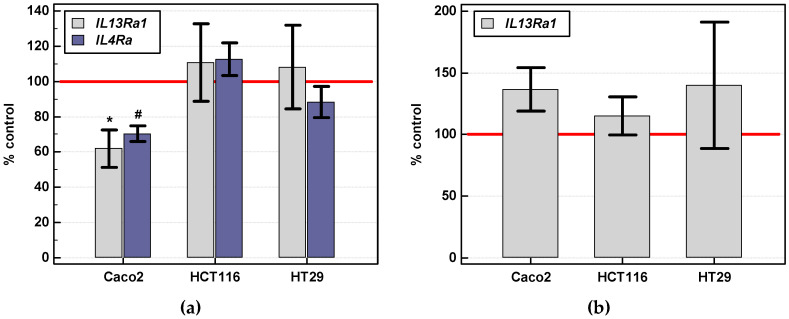
Effect of IL-4 and IL-13 on their respective receptor expression: (**a**) *IL13Ra1* and *IL4* expression following 24-h stimulation with 250 ng/mL of IL-4; (**b**) *IL13Ra1* expression following 24-h stimulation with 250 ng/mL of IL-13. Bars represent mean ± SE (*n* = 4) of relative expression of receptor transcripts in stimulated as compared to unstimulated (controls) cells expressed as a percentage. Reference expression level of untreated cells (100%) is represented by red horizontal line. Statistically significant differences in treated cells as compared to their respective controls, analyzed using *t*-test for one mean, are marked as follows: *p* < 0.05 as *; *p* < 0.01 as #.

*IL4Ra* and *IL13Ra1* expression in Caco-2 cells was significantly downregulated by 24-h stimulation with IL-4 (250 ng/mL). A minor non-significant upregulation of *IL4Ra* in HCT 116 and downregulation in HT-29 could be observed as well (Figure 12a). *IL13Ra1* expression was marginally upregulated in Caco-2 upon 24-h stimulation with IL-13 (250 ng/mL) but the effect did not reach statistical significance (Figure 12b).

#### 2.3.2. Effect of IL-4 and IL-13 on Viability of Colon Cancer Cells

Available literature presents contradicting data concerning effects of IL-4 and IL-13 on cancer cell proliferation and ability to evade pro-apoptotic signals. Therefore, we evaluated the interleukin impact on viability of colon cancer cells using sulforhodamine B (SRB) assay under normal growth conditions (cells cultured with 10% FBS) and under nutritional stress (cells cultured with 0.5% FBS). Subsequently, expression of genes encoding proliferation marker Ki67, anti-apoptotic proteins BCL-2 and BCL-xL, and cell cycle regulator p21^CIP1/WAF1^ in colon cancer cells stimulated with IL-4 or IL-13 was determined.

##### Viability Assay

**Figure 13:**
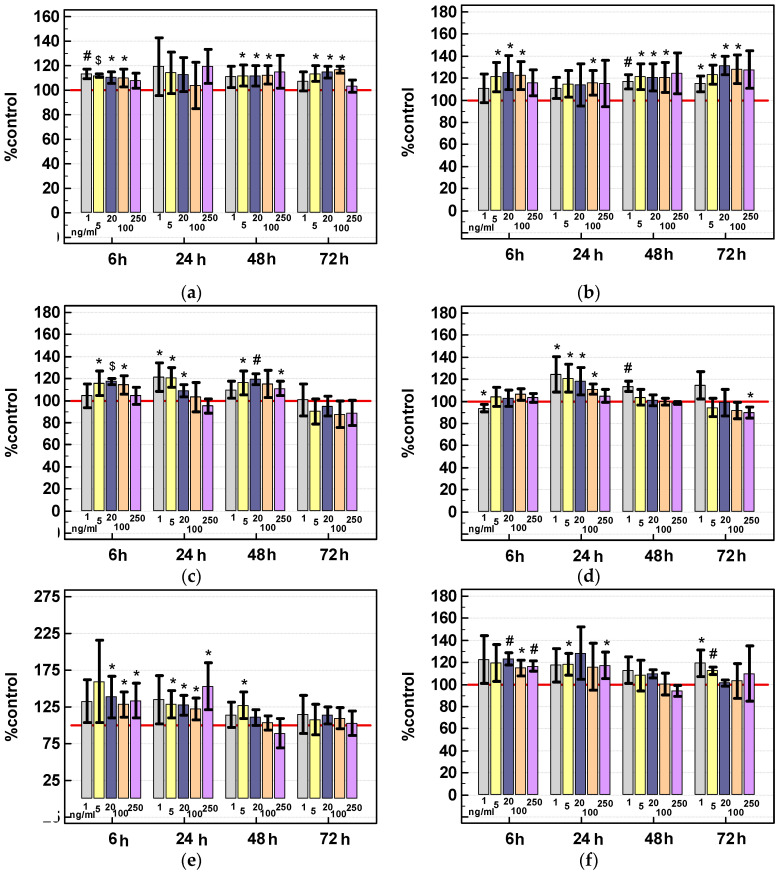
Effect of exogenous IL-4 (1–250 ng/mL) on cancer cell viability under normal (10% FBS) and nutritional-stress (0.5% FBS) conditions as determined with sulforhodamine B (SRB) assay: (**a**) HCT 116 cultured in 10% FBS; (**b**) HCT 116 cultured in 0.5% FBS; (**c**) HT-29 cultured in 10% FBS; (**d**) HT-29 cultured in 0.5% FBS; (**e**) Caco-2 cultured in 10% FBS; (**f**) Caco-2 cultured in 0.5% FBS. Data presented as percentage (% control) of viability of unstimulated cells, marked at 100% as reference red horizontal line. Statistically significant differences in treated cells as compared to their respective controls, analyzed using *t*-test for one mean, are marked as follows: *p* < 0.05 as *; *p* < 0.01 as #; *p* < 0.001 as $. Bars represent means ± SE of at least three experiments. FBS, fetal bovine serum.

Under normal growth conditions (10% FBS), IL-4 increased cell density by several to twenty percent in HCT 116 and HT-29 lines. Caco-2 cells response was less consistent but showed more marked growth acceleration (up-to 150% of control culture growth). Stimulatory effect of IL-4 was observed long-term in HCT 116 but short-term in Caco-2 cells. Moderate interleukin concentration, 5–100 ng/mL, was more effective while prolonged stimulation with 250 ng/mL of IL-4 tended to be inhibitory (Figure 13a,c,e).

Under nutritional stress conditions (0.5% FBS), stimulation with IL-4 resulted in accelerated growth in HCT 116 cells by 20–30%, especially in 72-h cultures, while the response of HT-29 and Caco-2 cells was weaker (Figure 13b,d,f).

**Figure 14:**
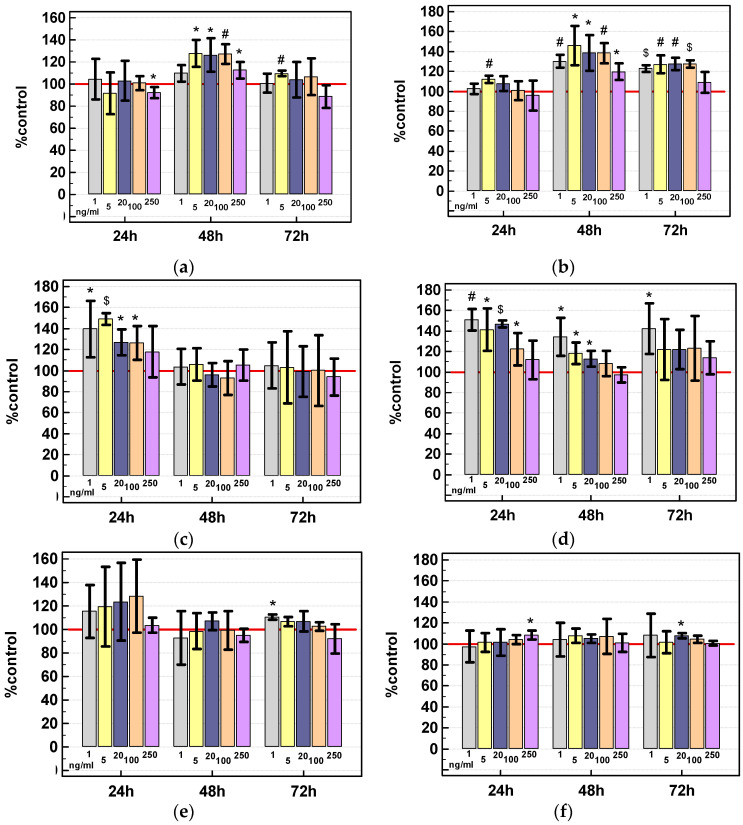
Effect of exogenous IL-13 (1–250 ng/mL) on cancer cell viability under normal (10% FBS) and nutritional-stress (0.5% FBS) conditions as determined with sulforhodamine B (SRB) assay: (**a**) HCT 116 cultured in 10% FBS; (**b**) HCT 116 cultured in 0.5% FBS; **(c**) HT-29 cultured in 10% FBS; (**d**) HT-29 cultured in 0.5% FBS; (**e**) Caco-2 cultured in 10% FBS; (**f**) Caco-2 cultured in 0.5% FBS. Data presented as percentage (% control) of viability of unstimulated cells, marked at 100% as reference red horizontal line. Statistically significant differences in treated cells as compared to their respective controls, analyzed using *t*-test for one mean, are marked as follows: *p* < 0.05 as *; *p* < 0.01 as #; *p* < 0.001 as $. Bars represent means ± SE of at least three experiments. FBS, fetal bovine serum.

Under normal growth conditions (10%FBS), IL-13 increased HCT 116 cell density by several to twenty five percent and the stimulatory effect was observed in 48-h cultures. The interleukin was more effective in HCT 116 cells grown under nutritional stress conditions (0.5% FBS). Cell density in 48-h cultures was higher by 40% and the stimulatory effect was observed in 48- and 72-h cultures. Short-term stimulation with IL-13 accelerated growth of HT-29 cells by 40%. Under nutritional stress conditions, the stimulatory effect of low interleukin concentration was observed in 48- and 72-h cultures. There was no or weak response to IL-13 in Caco-2 cells regardless the growth conditions (Figure 14).

##### Gene Expression

Increased cell viability may result from accelerated proliferation or increased resistance to apoptosis. Therefore, potential IL-4 and IL-13 effect on expression of genes encoding representative proteins relevant for either process was determined.

**Figure 15:**
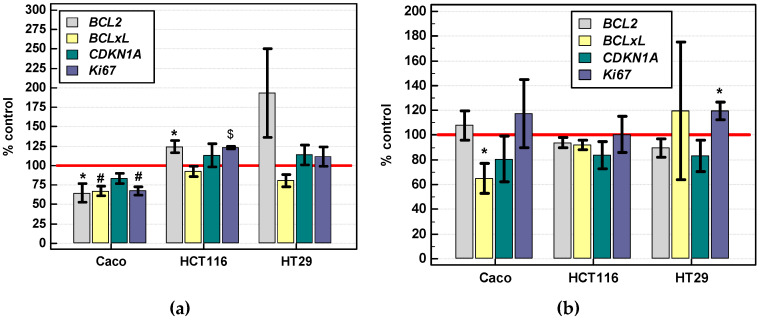
Effect of IL-4 and IL-13 stimulation on the expression of mediators of proliferation and apoptosis: (**a**) 24-h stimulation with 250 ng/mL of IL-4; (**b**) 24-h stimulation with 250 ng/mL of IL-13. Bars represent mean ± SE (*n* = 4) of relative gene expression in stimulated as compared to unstimulated (control) cells expressed as a percentage. Reference expression level of untreated cells (100%) is represented by red horizontal line. Statistically significant differences in treated cells as compared to their respective controls, analyzed using *t*-test for one mean, are marked as follows: *p* < 0.05 as *; *p* < 0.01 as #; *p* < 0.001 as $.

IL-4 stimulation had negative impact on Caco-2 cells, significantly decreasing the expression of *BCL2*, *BCLxL*, and *Ki67* and non-significantly that of *CDKN1A* (p21^CIP1/WAF1^). IL-4 slightly but significantly upregulated *BCL2* and *Ki67* expression in HCT 116 cells and more markedly *BCL2* expression, but the effect did not reach statistical significance (Figure 15a).

IL-13 stimulation significantly decreased *BCLxL* expression in Caco-2 cells and upregulated that of *Ki67* in HT-29 cells (Figure 15b).

#### 2.3.3. Effect of IL-4 and IL-13 on Motility of Colon Cancer Cells and Expression of Motility-Related Genes

IL-4 has recently been shown to enhance motility of colon cancer cells. Here, we evaluated the effect of IL-4 and IL-13 on cell motility using scratch assay. Subsequently, we analyzed the interleukin impact on genes encoding motility-related proteins: *ACTA2*, encoding EMT marker smooth muscle α-2 actin, and *TJP1* and *CLDN2*, encoding, respectively, tight junction proteins zonula occludens-1 and claudin-2.

##### Scratch Assay

Scratch assay was used to evaluate the effect of exogenous IL-4 and IL-13 (100 ng/mL) on the migratory properties of cancer cells recorded at 0, 24, 48, and 72 h.

**Figure 16:**
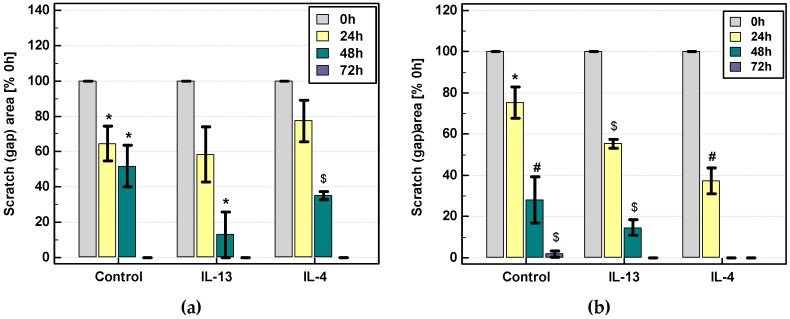
Analysis of the scratch assay data. Effect of IL-4 and IL-13 stimulation (100 ng/mL) on: (**a**) Caco-2 cells; (**b**) HCT 116 cells. Results at each time point are expressed as a percentage of gap area considering the gap at the time 0 as 100%. Each bar represents mean ± SE of three (Caco-2) or four (HCT116) biological replicates. Statistically significant differences as compared to time 0, analyzed using *t*-test for one mean, are marked as follows: *p* < 0.05 as *; *p* < 0.01 as #; *p* < 0.001 as $.

**Figure 17:**
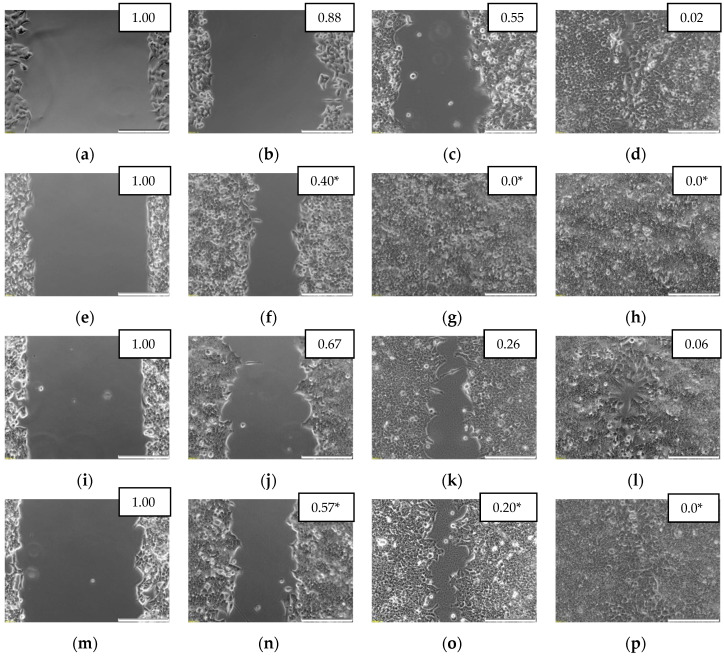
Impact of stimulation with 100 ng/mL of IL-4 or IL-13 on HCT 116 cells—a scratch assay (representative data): (**a**) control for IL-4 at 0 h; (**b**) control for IL-4 at 24 h; (**c**) control for IL-4 at 48 h; (**d**) control for IL-4 at 72 h; (**e**) stimulated with IL-4 at 0 h; (**f**) stimulated with IL-4 at 24 h; (**g**) stimulated with IL-4 at 48 h; (**h**) stimulated with IL-4 at 72 h; (**i**) control for IL-13 at 0h; (**j**) control for IL-13 at 24 h; (**k**) control for IL-13 at 48 h; (**l**) control for IL-13 at 72 h; (**m**) stimulated with IL-13 for 0 h; (**n**) stimulated with IL-13 at 24 h; (**o**) stimulated with IL-13 at 48 h; (**p**) stimulated with IL-13 at 72 h. Scale bars (200 µm) are indicated in lower right corner of each photograph. Numeric data in the upper right corner of each photograph indicate a ratio of gap (scratch) area at given time point to gap area at time 0. The ratios in interleukin-stimulated cells significantly (*p* < 0.01) different from their unstimulated counterparts are marked with asterisks (*).

**Figure 18:**
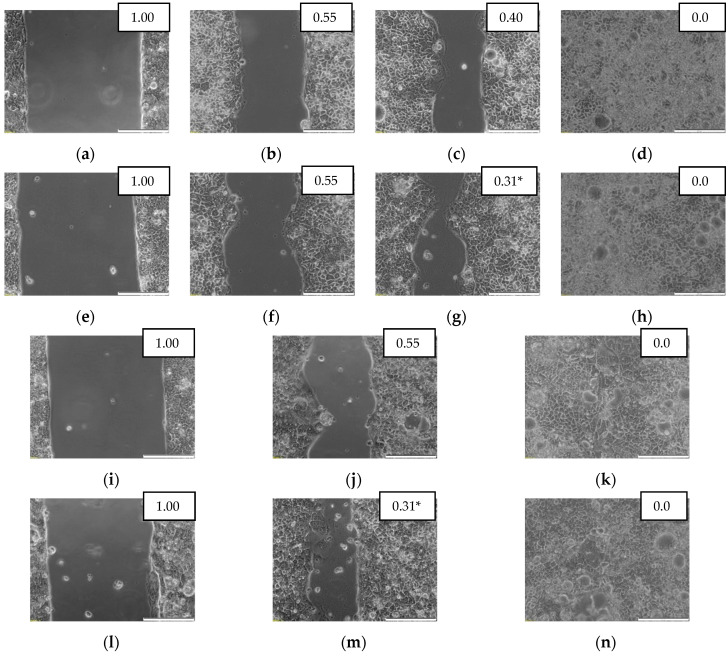
Impact of stimulation with 100 ng/mL of IL-4 or IL-13 on Caco-2 cells–a scratch assay (representative data): (**a**) control for IL-4 at 0 h; (**b**) control for IL-4 at 24 h; (**c**) control for IL-4 at 48 h; (**d**) control for IL-4 at 72 h; (**e**) stimulated with IL-4 at 0 h; (**f**) stimulated with IL-4 at 24 h; (**g**) stimulated with IL-4 at 48 h; (**h**) stimulated with IL-4 at 72 h; (**i**) control for IL-13 at 0 h; (**j**) control for IL-13 at 24 h; (**k**) control for IL-13 at 48 h; (**l**) stimulated with IL-13 at 0 h; (**m**) stimulated with IL-13 at 24 h; (**n**) stimulated with IL-13 at 48 h. Scale bars (200 µm) are indicated in lower right corner of each photograph. Numeric data in the upper right corner of each photograph indicate a ratio of gap (scratch) area at given time point to gap area at time 0. The rates in interleukin-stimulated cells significantly (*p* < 0.01) different from their unstimulated counterparts are marked with asterisks (*).

Both IL-4 and IL-13 stimulated motility of Caco-2 and HCT 116 cells. Analysis of data pooled from several biological experiments showed accelerated migration upon IL-4 and IL-13 stimulation at 48 h in Caco-2 and already at 24 h in HCT 116 cells. IL-13 seemed to be more effective than IL-4 in Caco-2 cells while IL-4 was more effective in HCT 116 (Figure 16). Photographs of exemplary scratch assay are depicted in Figure 17 for HCT 116 cells and in Figure 18 for Caco-2 cells. The migratory properties of HT-29 cells were generally low and the gap was not closed in control culture even after 144 h. Therefore, only IL-4 effect was tested and none was found.

##### Gene Expression

**Figure 19:**
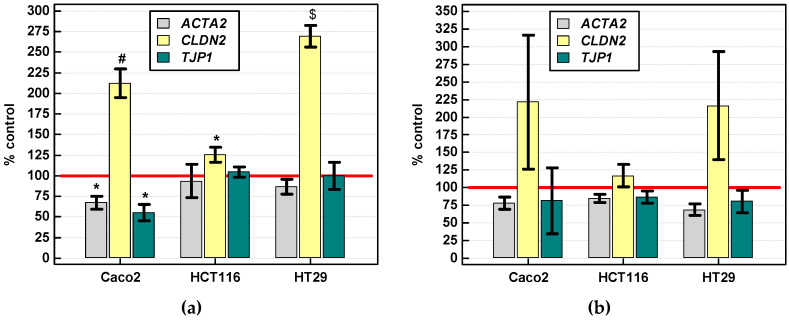
Effect of IL-4 and IL-13 stimulation on the expression of epithelial-mesenchymal transition markers and tight junction proteins: (**a**) 24-h stimulation with 250 ng/mL of IL-4; (**b**) 24-h stimulation with 250 ng/mL of IL-13. Bars represent mean ± SE (*n* = 4) of relative gene expression in stimulated as compared to unstimulated (controls) cells expressed as a percentage. Reference expression level of untreated cells (100%) is represented by red horizontal line. Statistically significant differences in treated cells as compared to their respective controls, analyzed using *t*-test for one mean, are marked as follows: *p* < 0.05 as *; *p* < 0.01 as #; *p* < 0.001 as $.

Stimulation with IL-4 (250 ng/mL) for 24-hs had consistently positive effect on *CLDN2* expression across all analyzed cell lines. In addition, IL-4 downregulated *ACT2* and *TJP1* expression in Caco-2 cells (Figure 19a). The expression of *CLDN2* was upregulated and those of *ACTA2* and *TJP1* downregulated in all cell lines following IL-13 stimulation (250 ng/mL, 24 h) but the effect did not reach statistical significance due to large variation between biological replicates of the experiment (Figure 19b).

#### 2.3.4. Effect of IL-4 and IL-13 on Expression of Various Cancer Development-Related Genes

**Figure 20:**
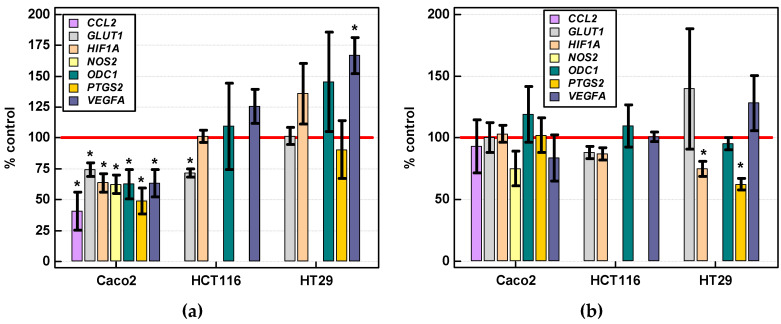
Effect of IL-4 and IL-13 stimulation on expression of various proteins relevant for cancer growth: (**a**) 24-h stimulation with 250 ng/mL of IL-4; (**b**) 24-h stimulation with 250 ng/mL of IL-13. Bars represent mean ± SE (*n* = 4) of relative gene expression in stimulated as compared to unstimulated (controls) cells expressed as a percentage. Reference expression level of untreated cells (100%) is represented by horizontal red line. Statistically significant differences (*p* < 0.05) in treated cells as compared to their respective controls, analyzed using *t*-test for one mean, are marked with asterisks (*).

Potential effect of IL-4 and IL-13 (250 ng/mL, 24 h) on the expression of genes encoding proteins relevant for cancer development by facilitating angiogenesis (*HIF1A* and *VEGFA*), inflammation (*PTGS2*, *NOS2*, *CCL2*), and metabolic reprogramming (*GLUT1*, *ODC1*, *NOS2*) was evaluated. IL-4 downregulated all assessed genes in Caco-2 cells and *GLUT1* in HCT 116 cells while upregulating *VEGFA* in HT-29 cells (Figure 20a). IL-13 significantly downregulated *HIF1A* and *PTGS2* in HT29 cells (Figure 20b). Expression of *CCL2* and *NOS2* in HCT 116 and HT-29 cells was too low to be reliably quantified. In turn, HCT 116 cells reportedly do not express *PTGS2* [32].

## 3. Discussion

The role of IL-4 and IL-13 in leukocyte biology and hematological malignancies is well established and their involvement in activation of cancer-promoting macrophages and myeloid-derived suppressor cells is increasingly being recognized [7,8]. However, it becomes apparent that both interleukins may directly affect the growth and progression of epithelial cancers if their cells express suitable receptors [7,8]. The presence of IL-4Rα protein has previously been demonstrated in the majority of investigated colonic [20,26] and gastric [25] tumors but data concerning esophageal cancer are scarce. Filling the gaps, we demonstrated that all examined tumor biopsies had detectable levels of receptor mRNA, including esophageal squamous cell carcinomas. More so, *IL4Ra* in esophageal tumors tended to increase along with growing cell dedifferentiation, apparently linking receptor expression with higher tumor aggressiveness. In CRC, however, *IL4Ra* expression was lower than in upper GIT tumors and was not associated with cancer pathology. Transcriptional analysis of clinical samples revealed positive correlation between *IL4Ra* and expression of negative regulators of apoptosis, markers of epithelial-mesenchymal transition, and mediators of inflammation and angiogenesis. While it might be viewed as an argument supporting pro-neoplastic character of *IL4Ra* expression, cellular composition of tumors is complex and our in vitro experiments showed that cancer cells specifically respond to cancer-promoting hypoxia with receptor down-regulation. Correspondingly, others have shown that high IL-4Rα protein expression in CRC was associated with lower incidence of lymph node metastasis [26]. Supporting the possible anti-neoplastic role for IL-4Rα, the experiments on knockout animals have revealed higher number of colorectal aberrant crypt foci following azoxymethane induction in animals lacking the receptor [27]. Nonetheless, functional studies regarding other epithelial cancers have shown IL-4Rα to facilitate the proliferation and survival of cancer cells at metastatic sites [33].

Likewise, *IL13Ra1* transcripts were detectable in all examined tumor samples and their expression level was the lowest in colonic samples, both cancerous and non-cancerous. Unlike the other IL-13 receptor, IL-13Rα2, data on IL-13Rα1 in the GIT cancers are limited and inconclusive. Previously, IL-13Rα1 protein expression has been demonstrated in the colon, in both adenomas [34] and adenocarcinomas [26,34]. However, immunoreactivity for IL-13Rα1 was markedly lower in colonic samples from patients with lymph node involvement, implying anti-neoplastic connotation for IL-13Rα1 expression [26]. In turn, the upregulation of IL-13Rα2 has been reported in colorectal [10] and gastric [16] cancers and linked with local advancement and metastasis [10] as well as poor prognosis [10,16]. Corroborating potentially tumor-promoting role also for IL-13Rα1, Matsui et al. [18] demonstrated co-expression of the receptor with proliferation indices in mice with obesity-related CRC. Moreover, the authors showed an inhibition of colonic cancer cell proliferation induced by IL-13 by silencing *IL13Ra1,* but not *IL13Ra2* gene. We, in turn, observed *IL13Ra1* in tumors to correlate positively with expression of genes encoding proteins involved in promoting cancer, especially those facilitating invasion and metastasis such as α smooth muscle actin, HIF1α, and VEGF-A, although not with proliferation marker Ki67. Like *IL4Ra*, however, *IL13Ra1* expression in colonic cancer cells was uniformly downregulated under hypoxia.

Clinical data on IL-4, a primary ligand for IL-4Rα, are equivocal. Here, IL-4 protein was upregulated at both local and systemic level and regardless the anatomical site. However, while the local protein concentration was the highest in CRC and the lowest in GC, circulating IL-4 was more markedly elevated in GC than CRC. This finding might imply a more immunosuppressive tumor microenvironment in CRC and provide rationale for its poor responsiveness to checkpoint blockade, the otherwise successful antitumor strategy based on alleviating tumor-induced inhibition of adaptive immunity [35]. Nonetheless, it has been put forward that systemic elevation of Th2 cytokines, observed here particularly in gastric cancer, may indicate a generalized state of immunosuppression and still promote cancer growth and dissemination [36]. Different association patterns for local and systemic IL-4, concerning sex-dependence [15,37] and relation with the disease advancement [13,14], have been noted also by other authors. Regarding CRC, previous studies have shown circulating IL-4 to be too low to be quantified [38] or, in contrary, elevated [39,40,41] and suitable as a CRC biomarker. Moreover, in favor of its cancer-supporting role, IL-4 elevation has been directly linked with higher mortality [42]. In addition, interleukin concentration has been increased in right-sided colonic cancers [39], considered more aggressive and associated with worse prognosis [43,44,45]. Furthermore, the interleukin over-secretion has been confirmed already in conditions associated with increased risk of CRC, that is, in ulcerative colitis [46] and adenomatous polyps [41]. Unlike in CRC or esophageal cancer [47,48], an elevation in systemic IL-4 has been unanimously reported in GC [37,47,49], even though its association with pathological findings was inconsistent. On the one hand, IL-4 has been more markedly elevated in patients with higher modified Glasgow Prognostic Score, thus with worse prognosis, but lower in those with more advanced cancers, that is, with vascular invasion or N3 cancers, on the other [49].

Contrary to systemic interleukin concentration, its local protein level in our patients depended on tumor aggressiveness and cancer advancement in ESCC and GC patients, respectively. Interestingly, however, only in GC the correlation resulted from IL-4 accumulation in tumor. In ESCC, the increasing difference in interleukin concentration between tumor and non-cancerous tissue stem from its decrease in adjacent tissue, inversely proportional to tumor grade, implying a protective role for the interleukin. Likewise, IL-4 protein concentration in colonic non-cancerous tumor-adjacent tissue decreases along with increasing tumor grade. Although Todaro et al. [12] suggested a possibility of autocrine mode of IL-4 signaling, we found that *IL4* transcripts were detectable exclusively in one out of six investigated cell lines and their expression level was low. It would suggest that interleukin downregulation in adjacent tissue is not contributed to tumor cells. More likely, it is associated with its decreased secretion by other cell types and/or reflects alterations in cellular composition, with reduced number of IL-4 secreting cells. In line with declining IL-4 concentration along with increasing cancer aggressiveness in ESCC and CRC, *IL4* expression was lower in ESCC cancers metastasizing to lymph nodes and tended to be inversely correlated with primary tumor extension in CRC. In turn, consistently with data regarding interleukin concentration, *IL4* expression in gastric tumors tended to increase with the disease advancement. Whether IL-4 plays a protective or cancer-promoting role in GIT carcinogenesis is controversial. Supporting notion on protective function, lower IL-4 immunoreactivity has been demonstrated in colorectal tumors from CRC patients with lymph node metastases [26] or gastric tumors from patients with stage III/IV cancers [15]. In addition, IL-4 immunoreactivity inversely correlated with the presence of gastric metaplasia or cancer and with degree of inflammation [14]. Corroborating the latter observation, the expression of *IL4* transcripts in our clinical samples correlated negatively with inducible nitric oxide synthase, an enzyme involved in inflammatory response and oxidative stress. Yet, the association was found solely in tumors while in non-cancerous tissue *IL4* positively correlated with the expression of cyclooxygenase 2 (COX2) and monocyte chemoattractant protein 1 (MCP-1). Regarding MCP-1, IL-4 and MCP-1 are reportedly involved in a “self-amplifying loop” in tumor microenvironment, whereby interleukin promotes MCP1 expression by endothelial cells, which, in turn, induces Th2 differentiation by up-regulating IL-4 synthesis [50]. Caco-2 cells, however, responded to IL-4 stimulation with downregulation of *NOS2* and COX2 and MCP1 encoding genes, *PTGS2* and *CCL2*. Likewise, IL-4 downregulated the expression of *PTGS2* in another epithelial cancer, that is, non-small cell lung cancer cells [51]. Taking into account a wide spectrum of cancer-promoting activities of MCP-1 or COX2, the downregulation of their expression induced by IL-4 seem to support anti-neoplastic character of the interleukin. Still, others have shown that IL-4 may initiate the carcinogenesis by triggering the premalignant differentiation of gastric epithelia [19] or by reducing squamous cell markers in the esophagus and inducing columnar differentiation with expression of columnar markers (reviewed in [21]). In the colon, IL-4 has stimulated invasion and metastasis by promoting the epithelial-mesenchymal transition via activation of STAT6 and transcriptional repressors Zeb [52].

While GC patients had the highest circulating IL-4, they had also the lowest concentration of IL-13, significantly lower even than enrolled controls. Elevated systemic IL-13 reflected the disease advancement in ESCC, particularly the presence of lymph node metastasis and the depth of tumor invasion, and tended to correlate positively with cancer stage and tumor grade in CRC. Fold-change in IL-13 protein concentration in the colorectum correlated positively with lymph node involvement and the disease stage. Like IL-4, IL-13 dropped in non-cancerous tissue. Contrary to IL-4, however, IL-13 protein concentration in esophageal cancerous and non-cancerous tissue increased along with ESCC advancement while in gastric tissue—decreased. Also differently than observed for IL-4, IL-13 protein concentration and *IL13* transcript expression displayed different association patterns with pathological findings. *IL13* in CRC correlated inversely with lymph node metastasis and TNM due to its increasing expression in non-cancerous tissue, paralleling increasing cancer advancement. Available literature on IL-13 in the GIT cancers is even scantier than that concerning IL-4. In CRC, high systemic IL-13 translated into better prognosis and accompanied less advanced cancers in terms of the overall disease stage and lymph node and distant metastases [24]. In ESCC, the density of IL-13 protein in tumor stroma has been higher in early cancers and positively associated with overall and disease-free survival [23]. Correspondingly, *IL13* in clinical samples was inversely related to the expression of *GLUT1*, the upregulation of which is a hallmark of cancer-related metabolic reprogramming. In addition, it correlated negatively with anti-apoptotic *BCL2* and proinflammatory *NOS2*, corroborating notion on beneficial interleukin role. Stimulation with IL-13 downregulated *PTGS2* expression in HT-29 cells and reduced that of *HIF1A*, although *IL13* in clinical samples was correlated positively with *VEGFA*, a HIF1α-inducible key mediator of angiogenesis. Nonetheless, contradicting the implied protective role [23], animal studies have implicated IL-13 in the development of metaplasia during gastric carcinogenesis (reviewed in [22]) and obesity-related CRC [18] while our own results have shown systemic interleukin to be elevated in both CRC [39] and high-risk conditions [46].

One of the important findings of this study is the observation that some of the alterations concerning interleukins are happening in the tumor-adjacent tissue and not the tumor itself. There is growing awareness that the macroscopically normal tumor-surrounding tissue may be changed at molecular level. While insufficient to affect cell morphology, it may predispose to neoplastic transformation. This phenomenon is believed to contribute to synchronous multiple cancers and to the disease recurrence following tumor resection. Curative resection remains the mainstay in treatment of gastrointestinal cancers. Resection margins are assessed histopathologically for the presence of cancer cells while their concomitant evaluation at molecular level is argued to provide more accurate information on recurrence risk and thus facilitate the clinical decision-making [53,54]. It has been pointed out [55] that unraveling molecular alterations in still non-transformed, tumor-adjacent tissue may allow for better understanding of the processes leading to malignancy than the analysis of changes occurring in already transformed cells. In further perspective, it may facilitate developing strategies for their earlier detection and for primary chemoprevention [55]. As we have encountered also in our earlier research [56,57,58], the exclusive analysis of expression ratio might be inaccurate because of tumor and adjacent tissue being similarly affected. Here, it was best exemplified by lack of association between fold-change in IL-13 protein concentration and ESCC or GC pathology as interleukin level changed along with the disease advancement to similar degree in cancerous and non-cancerous tissue.

The analysis of interleukin expression separately in tumor and adjacent tissue allowed us to demonstrate also the differences with respect to the anatomical sites. Higher levels of *IL4*, *IL4Ra,* and *IL13Ra1* mRNA expression were detected in gastric than colorectal cancer, which, taking into account their association with tumor progression and aggressiveness may contribute to worse prognosis associated with gastric cancers. As compared to colorectal cancers, both gastric and esophageal cancers have substantially shorter survival rates and higher prevalence of poorly differentiated cancers (G3) [59]. Still, the observations made at mRNA level were in opposition to those made for IL-4 protein, as its protein concentration was significantly higher in CRC than GC, both for tumor and adjacent tissue. In turn, *IL13* mRNA was lower in GC than CRC tumors while IL-13 protein was more markedly expressed in GC than CRC. It has been pointed out [60] that lack of association, or poor correlation, between transcript number and protein abundance is quite a common occurrence. It has been attributed to a complicated process of protein translation followed by various modifications to synthesized proteins as well as to the substantial differences in mRNA and protein half-life, frequently leading to protein accumulation while respective transcripts have been degraded. Therefore, treating data on mRNA and protein as separate information sources has been encouraged [60].

As there is no consensus concerning interleukins effect on cancer cells in vitro, we assessed their impact on two basic cell activities, that is, viability (net result of proliferation and survival) and mobility. We found IL-4 and IL-13 to improve both; yet, to different degrees, depending on colonic cell line. Others, however, have shown HT-29 cells to respond to IL-4 with reduced proliferation [28,29]. Of note, an anti-proliferative activity has been demonstrated also in gastric [30] and other epithelial cancers [29,61,62]. Still, Liu et al. [17] demonstrated that both IL-4 and IL-13 increase colon cell proliferation via upregulating expression of NADPH oxidase (NOX)-1 and generation of reactive oxygen species. A pro-proliferative activity of IL-4 toward HT-29 and HCT 116 cells was reported by Keller et al. [20]. In turn, pro-proliferative activity of IL-13 toward HT-29 was recently corroborated by Matsui et al. [18]. The results of Chang et al. [28] implicated that the dual IL-4 effect on proliferation might depend on whether the interleukin activates STAT1 (anti-proliferative) or STAT6 (pro-proliferative) as previously found in helper T cells [63]. Others, however, have shown IL4-induced STAT6 activation to be involved in growth inhibition [61]. In this study, growth-promoting activity of IL-4 was observed in HT-29 as well as HCT 116 and Caco-2, but the interleukin increased cell density only by several to twenty percent. Moreover, the stimulatory effect in HT-29 and Caco-2 could be observed after short incubation, like reported by Koller et al. [20], who stimulated cells for 24 h. Prolonged treatment, in turn, especially with high interleukin concentration, tended to be associated with growth inhibition, as demonstrated during 3-day stimulation by Chang et al. [28]. The SRB assay used in this study determines the protein content and therefore the cell density, which might indicate the accelerated proliferation or reduced apoptosis or being the result of both. Here, only *IL4Ra* correlated with the proliferation marker Ki67 in clinical samples but the association was negative. Negative effect on Ki67 expression had also a stimulation of Caco-2 cells with IL-4. Still, the marker was upregulated, although weakly, in HCT 116 cells stimulated with IL-4 and HT-29 cells stimulated with IL-13. IL-4 is reportedly conferring cancer cells protection against apoptosis. Neutralizing antibodies to IL-4 have inhibited tumor growth in xenograft models by reducing expression of anti-apoptotic Bcl-xL and Bcl-2 [12]. Anti-apoptotic activity of IL-4 has been confirmed also in thyroid [64], lung, and breast cancer [12]. Importantly, anti-apoptotic activity of IL-4 has been associated with an autocrine mode of action [12]. As already mentioned, the cell lines tested did not express *IL4* or *IL13*. Therefore, it may contribute to mostly negative impact of IL-4 or IL-13 on *BCL2* and *BCLxL* expression.

The similar results of growth promotion were obtained in this study when cancer cells were treated with IL-4 under nutritional stress conditions. The metabolic reprogramming, a recently recognized hallmark of cancer [5], ensures growth advantage over non-transformed and immune cells and promotes cancer cell survival. IL-4 is known to accelerate glucose and glutamine up-take and enhance their metabolism in immune cells and has recently been demonstrated to partake in metabolic reprogramming of breast cancer cells as well [33]. However, our results do not support IL-4 or IL-13 contribution to metabolic reprogramming in CRC. The expression of glucose transporter *GLUT1* was rather downregulated in Caco-2 and HCT 116 cells stimulated with IL-4 and its expression in clinical samples was negatively correlated with *IL13*.

Koller et al. [20] reported that pro-proliferative effects are dominant for IL-4 stimulation of colon cancer cells as only HCT 116 and neither HT-29 nor SW620 responded to IL-4 with enhanced survival. The growth dynamics observed in our study, particularly the difference between HCT 116 and Caco-2 or HT-29 cells at 72-h incubation, might therefore be explained by the weakening of the pro-proliferative effect with prolonged stimulation and, exclusively in HCT 116, by line response to pro-survival activity of IL-4. Especially, that growth-stimulating effect of the interleukin on HCT 116 cells was slightly stronger in cells grown under nutritional stress conditions. Interestingly, the growth-supporting effect on HCT 116 cells under nutritional stress conditions was stronger for IL-13 than IL-4. Both interleukins may signal through the same type II IL-4 receptor and elicit similar responses, like was the case in present study, although the sensitivity of examined cell lines differed. In addition to already mentioned slightly greater sensitivity of HCT 116 to pro-survival activity of IL-13 during prolonged stimulation, the migratory properties of those cells were more markedly improved by IL-4. Caco-2 cells, in turn, seemed to respond better to IL-13. Of note, Caco-2 response to IL-4 seem to differ from that observed in HCT 116 and HT-29 also concerning *BCL2* and *Ki67* expression, down-regulated in Caco-2 but slightly upregulated in HCT 116 and HT-29. Moreover, unlike in immune cells [31], IL-4 stimulation downregulated its receptor expression in Caco-2 cells but not HCT 116 and HT-29. Dissimilar responses might be partly explained by different expression level of IL-4 receptor. Indeed, Caco-2 had significantly higher *IL4Ra* expression than HCT 116 cells.

As mentioned earlier, Chen et al. [52] implicated E2F1/SP3/STAT6 axis and transcription factors Zeb in the epithelial-mesenchymal transition induced by IL-4. Here, we evaluated IL-4 and IL-13 effect on genes encoding mesenchymal marker, *ACTA2*, and tight junction proteins ZO-1 (*TJP1*) (epithelial marker) and claudin-2 (*CLDN2*). Tight junction proteins preserve cell adhesiveness and prevent dissociation of tumor cells and thus cancer metastasis. Their aberrant expression patterns have been repeatedly noted in epithelial cancers [65]. Disruptive properties of IL-4 and IL-13 on epithelial barrier integrity have been reported. Specifically, the interleukins have been shown to upregulate claudin-2 and downregulate ZO-1 in colonic cancer cell line T84 and airway epithelium, respectively [66,67]. Correspondingly, we found that colonic cancer cells responded to IL-4 and IL-13 stimulation with upregulation of *CLDN2*, significantly so in case of IL-4, while *ACTA2* and *TJP1* tended to be rather downregulated, significantly so in case of Caco-2 stimulation with IL-4. However, in clinical samples, heterogeneous with respect to cellular composition, interleukin receptor expression was positively correlated with both *ACTA2* and *TJP1*.

Our study has several limitations that need to be addressed. First, we aimed at quantitative analysis and used appropriate methodology (immunoassays and RTqPCR), which, however, does not allow for indicating cellular origins. As such, lack of complementary immunohistochemistry data may be viewed as a limitation. Second, the in vitro part of our study has an exploratory character aimed at screening for potential associations to select points of interest for future in-depth functional studies discerning signaling pathways involved. As the screening was conducted using transcriptional analysis, our results ought to be confirmed on protein level.

## 4. Materials and Methods

### 4.1. Patients

#### 4.1.1. Study Population for Analysis of Local Interleukin Expression

The study population consisted of 49 (for protein determination) or 51 (for transcriptional analysis) cancer patients, admitted to the Department of Gastrointestinal and General Surgery of Wroclaw Medical University for curative resection of esophageal squamous cell carcinomas (*n* = 18/16) or gastric (*n* = 14/14) or colorectal (*n* = 17/21) adenocarcinomas. Patients with any severe systemic illness, with gross metastatic disease, or subjected to radio- or chemotherapy were not included. Patients were subjected to standard preoperative evaluation (blood work, physical examination, and imaging techniques, such as ultrasonography, computed tomography and magnetic resonance). Cancers were rated pathologically using 7th edition of the Union for International Cancer Control TNM system. In all cases, the resection margins have been confirmed to be tumor-free. Detailed population characteristics are depicted in Table 1 and Table 2.

#### 4.1.2. Study Population for Determination of Systemic Interleukin Concentration

The study population consisted of 263 individuals: 36 controls and 93 patients with histologically confirmed squamous cell carcinomas of the esophagus, 31 with histologically confirmed adenocarcinomas of the stomach, 32 with histologically confirmed adenocarcinomas of the gastric cardia, and 71 with histologically confirmed adenocarcinomas of the colorectum. All patients were admitted to the Department of Gastrointestinal and General Surgery of Wroclaw Medical University for the disease diagnosis and/or treatment (curative surgery or palliative treatment). Cancers were rated clinically using 7th edition of the Union for International Cancer Control TNM system. Healthy individuals (*n* = 36) were recruited from otherwise healthy outpatients of Research, Science, and Educational Center of Dementia Diseases, Scinawa, Poland suffering from headaches (dementia, neurological disorders, and brain tumors were excluded during diagnostic process) and from blood donors whose sera were provided by the Regional Center of Blood Donation and Therapeutics in Wroclaw, Poland. Detailed population characteristics are depicted in Table 3.

### 4.2. Ethical Considerations

The study protocol was approved by the Medical Ethics Committee of Wroclaw Medical University (signature number: KB 203/2016). The study was conducted in accordance with the Helsinki Declaration of 1975, as revised in 1983, and informed consent was obtained from all study participants.

### 4.3. Analytical Methods

#### 4.3.1. Local Interleukin Concentration (Protein)

Patient-matched pairs of postoperatively collected tissue samples from the tumor and from the macroscopically normal tissue adjacent to the tumor (taken approximately 10 cm from the tumor) were rinsed with PBS, rapidly frozen and stored at −45 °C until analysis.

Weighted 10-40 mg fragments were homogenized (2 min, 4.0 m/s) in 10 mM Tris-HCl buffer with 150 mM KCl and 1 mM EDTA, pH 7.4 in proportion 1:2 (*w/v*) with ceramic spheres and FastPrep-24 homogenizer (MP Biomedical, Solon, OH, USA). Tissue homogenates were centrifuged (14,500× *g*, 10 min, 6 °C) and the resulting supernatants were used for interleukin determination.

IL-4 and IL-13 concentrations in clarified tissue homogenates were measured immunoenzymatically using respective Quantikine high-sensitive (HS) ELISA assays for human IL-4 and IL-13, purchased from RnD Systems (Minneapolis, MN, USA). The assays were performed according to manufacturers’ instructions. All measurements were conducted in duplicates. Data are expressed as pg (IL-4) or ng (IL-13) of protein per gram of analyzed tissue.

#### 4.3.2. Systemic Interleukin Concentration

Peripheral blood was collected into BD Vacutainer CAT tubes (Becton Dickinson, Plymouth, UK), clotted for 30 min at room temperature and subsequently centrifuged at 1500× *g* for 10 min at room temperature. Obtained sera were aliquoted and stored at −45 °C until examination. Blood samples were collected upon admission, prior to any treatment, following overnight fast.

Serum concentrations of IL-4 and IL-13 were measured using immunofluorescence on the BioPlex 200 platform with HRF (Bio-Rad, Hercules, CA, USA), incorporating Luminex xMAP^®^ technology, and Bio-Plex Pro™ Human Cytokine, Chemokine, and Growth Factor Magnetic Bead–Based Assays. The method is based on flow cytometry. It utilizes magnetic microspheres conjugated with monoclonal antibodies. All analyses were conducted in duplicates and according to the manufacturer’s instructions. Standard curves were drawn using 5-PL logistic regression and the data were analyzed using BioPlex Manager 6.0 software.

#### 4.3.3. Transcriptomic Analysis

Intraoperatively collected tissue specimens (paired, patient-matched tumor and macroscopically normal samples) were rinsed with PBS, soaked in RNAlater (Ambion Inc., Austin, TX, USA) and stored at −80 °C until RNA isolation.

Tissue samples (up-to 40 mg) were homogenized using Fastprep 24 Homogenizer (MP Biomedical, Solon, OH, USA) in lysis buffer (provided as a part of PureLink™ RNA Mini Kit) with addition of β-mercaptoethanol (Sigma-Aldrich, St. Luis, MO, USA).

Upon termination of cell culture experiments, cells were scratched and lysed with 1 mL of TRIzol Reagent (Thermo-Fisher Scientific, Waltham, MA, USA) and stored at −80 °C until RNA isolation.

RNA from tissue samples and cell cultures was isolated using phenol-chloroform extraction and subsequently purified using PureLink™ RNA Mini Kit (Thermo-Fisher Scientific). Genomic DNA was removed by on-column treatment of RNA isolates with DNase (PureLink™ DNase Set, Thermo-Fisher Scientific). The concentration of isolated RNA was quantified using NanoDrop 2000 (Thermo-Fisher Scientific) with concomitant evaluation of RNA purity (ratios of absorbance at 260, 280, and 230 nm). RNA integrity was evaluated using the Experion platform incorporating LabChip microfluidic technology and Experion RNA StdSens analysis kits (BioRad) and expressed as RNA quality indicator (RQI) score with RQI=1 indicative of degraded and RQI = 10 of intact RNA. Only RNA isolates with RQI ≥ 7 were used for reversely transcribed quantitative polymerize chain reaction (RT-qPCR).

The 1000 ng of RNA per reaction mixture (20 µL) was reversely transcribed in C1000 termocycler (BioRad) using iScript™ cDNA Synthesis Kit (BioRad) and the protocol suggested by manufacturer. Samples were accompanied by matching negative transcription (“no-RT”) controls, devoid of reverse transcriptase, subsequently tested to assure lack of contamination with genomic DNA.

Quantitative PCRs were conducted using CFX96 Real-Time PCR system (BioRad) and SsoFast EvaGreen^®^ Supermix (BioRad). The following cycling conditions were applied: 30 s activation at 95 °C, 5 s denaturation at 95 °C, annealing/extension for 5 s at 61 °C, 40 cycles, followed by melting step (60–95°C with fluorescent reading every 0.5 °C). Reaction mixture contained 2 µL of cDNA (diluted 1:5), 10 µL of 2 × SsoFast EvaGreen^®^ Supermix, 1 µL of each 10 nM forward and reverse target-specific primers, and water up to 20 µL. Only primers spanning at least one intron were used. Their specificities were tested by melting curve analysis and an electrophoresis in a high-resolution agarose (SeaKem LE agarose from Lonza, Basel, Switzerland) in TBE with SYBR Green (Lonza) detection. Only primers yielding a single peak in melt curve and a single band in gel electrophoresis were used. Primers were also tested on no–RT samples with known contamination with gDNA and only these not amplifying gDNA at all or with no–RT signal at least 10 cycles apart from sample signal were selected. Primers were synthesized by Genomed (Warsaw, Poland) and their sequences are presented in Table 12.

Technical replicates were averaged prior analysis. Geometric mean of all Cq values in a given analysis was obtained and subtracted from sample Cq (ΔCq) then linearized by 2^^ΔCq^ conversion and normalized to *GAPDH*. The obtained values are referred to as a normalized relative quantity (NRQ) [68] and subjected to statistical analysis. For paired sample analysis, ΔCq values obtained for normal and pathological tissue or treated and untreated cells were subtracted (ΔΔCq) and the relative level of expression (fold change in expression) was calculated as 2^^(-ΔΔCq)^.

#### 4.3.4. Cell Cultures and Cell Culture Experiments

Certified human adherent colon cancer cell lines: Caco-2 (ATCC^®^ HTB-37™), HCT 116 (ATCC^®^ CCL-247™) and HT-29 (ATCC^®^ HTB-38™) were purchased from ATCC (MD, USA). Cells were cultured in 75 cm^2^ cell culture flasks (Nunc™ EasYFlask™ Nunclon™ Delta Surface; Thermo Fisher Scientific, Waltham, MA, USA) at 37 °C in 95% air with 5% CO_2_ in CELCULTURE^®^ CCL-170B-8 incubator (Esco, Singapore), in Dulbecco’s modified eagle medium (DMEM; Gibco, Thermo Fisher Scientific), supplemented with 10% fetal bovine serum (Gibco), 1% GlutaMAX™-I (Gibco), and with 1% stabilized antibiotic antimycotic solution (10,000 units penicillin/mL, 10 mg streptomycin/mL, 25 µg amphotericin B/mL; Sigma Aldrich, St. Louis, MO, USA). Medium was renewed in every 3 days. Cells were harvested with TrypLE™ Express (Gibco) after rinsing with Dulbecco’s Phosphate Buffered Saline (DPBS; Gibco). Cells were counted with Countess™ Automated Cell Counter (Invitrogen, Thermo Fisher Scientific, Waltham, CA, USA) after staining with 0.4% trypan blue solution (Invitrogen).

##### Sulforhodamine B (SRB) Viability Assay

Cells were seeded at 1.5–2 × 10^4^ cells/well on a 96-well plate in three to five technical replicates. For specified times (6 (only for IL-4), 24, 48, or 72 h), cells were treated with recombinant human IL-4 or IL-13 (RnD Systems, Minneapolis, MN, USA) in culture medium at concentration of 0 (control) 5, 20, 100, or 250 ng/mL. The experiments were conducted under normal growth conditions (medium supplemented with 10% FBS) and under nutritional stress (medium supplemented with 0.5% FBS). After specified incubation time, cells were fixed by gentle addition of cold trichloroacetic acid (Sigma-Aldrich) directly into culture medium to the final concentration of 12.5%. After 1-h incubation at 4 °C, plates were washed with cold water, dried and 0.04% SRB in 1% acetic acid solution (Sigma-Aldrich) was added for 30-min incubation at room temperature. The unbound dye was rinsed with 1% acetic acid while the protein-bound SRB was solubilized by addition of 10 mM Tris base solution (pH 10.5). The absorbance, proportional to protein content, was measured at 492 nm using Infinite M200 plate spectrophotometer (Tecan Group Ltd., Männedorf, Switzerland). The experiments were conducted at least three times.

##### Scratch Assay

The 70 µL of cell suspension containing 5 × 10^4^ cells was applied into each well of the Culture-Insert 2 Well (placed in 24-well cell culture plate; Ibidi, Gräfelfing, Germany) and cultured for 24 h. Subsequently, the inserts were removed from wells and wells were rinsed with DPBS, followed by the addition of medium without or with 100 ng/mL of IL-4 or IL-13. The closure of insert-created gap (wound) was observed in three (Caco-2) or four (HCT 116) independent experiments after 24, 48, and 72 h under the CKX41 inverted microscope with SC30 camera (Olympus, Tokyo, Japan). For HT-29 cells, the experiment was conducted up-to 144 h and exclusively for IL-4. Gap area was measured using the MRI Wound Healing Tool macro for ImageJ software (NIH) [69]. Subsequently, the ratios of measured gap areas were calculated (area at a given time point/area at time 0) and statistically compared between stimulated and unstimulated cells. Additionally, mean gap areas (expressed as a percent of gap area at time 0 h) were plotted against time.

##### Cell Culturing for Transcriptional Analysis

For gene expression analysis, 2 × 10^5^ cells/well were seeded on plastic, flat bottom, 6-well plates (Corning CellBIND surface) and cultured at 37 °C in a humidified atmosphere containing 5% CO_2_ until 80% confluence. Subsequently, in serum withdrawal experiment, the complete medium (with 10% FBS) was replaced with a fresh one with low FBS concentration (0.5%)-except for control cells (serum-supplemented)-and cells were grown for 24 h. In chemically induced hypoxia experiment, the complete medium was replaced with a fresh one containing 200 µM CoCl_2_ (Sigma-Aldrich) for 24 h and in the experiments evaluating the effect of IL-4 or IL-13 stimulation, medium was replaced for 24 h by a fresh one containing interleukins at 250 ng/mL concentration. Following medium removal, treated and untreated (control) cells were lysed with Trizol (Thermo-Fisher Scientific) and stored at −80 °C until RNA isolation.

#### 4.3.5. Statistical Analysis

Data were tested for normality of distribution and homogeneity of variances using Kolmogorov-Smirnov test and Levene test, respectively. Paired data were analyzed using *t*-test for paired samples. Two-group comparisons were conducted using *t*-test for independent samples, with Welch correction in case of unequal variances. Resulting data are presented as means/geometric means with 95% confidence interval (*CI*). Multigroup comparisons were conducted using one-way ANOVA, following log-transformation if suitable, with Tukey-Kramer post-hoc test or Kruskal-Wallis *H* test with Conover post-hoc test. Resulting data are presented as, respectively, geometric means or medians with 95% *CI*. The *t*-test for one mean and comparison of rates were used to analyze in vitro data. Frequency analysis was conducted using χ^2^ test while correlation analysis using Spearman’s rank correlation test (ρ) or Pearson correlation (r) as indicated. All calculated probabilities were two-tailed. The *p* values ≤ 0.05 were considered statistically significant. The entire analysis was conducted using MedCalc Statistical Software version 19.2 (MedCalc Software Ltd., Ostend, Belgium; https://www.medcalc.org; 2020).

## 5. Conclusions

Colorectal as well as upper gastrointestinal tract tumors have higher concentration of IL-4 and IL-13 than adjacent non-cancerous tissue, which, solely in CRC tumors, is accompanied by elevated expression of *IL13* transcripts. Local expression of ILs and their receptors reflect cancer pathology in a cancer type-depended manner. Specifically, the elevation of IL-4 in gastric tumors (protein and mRNA) is directly proportional to the disease advancement. In ESCC and CRC, in turn, the more advanced and aggressive disease, the less IL-4 protein and mRNA are present and the alterations occur in non-cancerous adjacent tissue rather than in tumor. The observation concerning differences in expression levels with respect to the anatomical site as well as to whether they concern protein or mRNA transcripts ought to be taken into account while planning immunotherapies affecting IL-4/IL-4R and IL-13/IL-13R pathways. Finding on altered IL/ILR expression in non-cancerous tissue adds to the increasing knowledge on tumor molecular margin and is potentially relevant for clinical decision-making.

We found that receptor expression in colonic cell lines is negatively affected by hypoxia, what seems to support antiangiogenic role of IL-4/IL4R axis. Still, receptor expression in clinical tumor samples is correlated positively with a number of genes encoding proteins relevant for cancer development and progression. However, with few exceptions, observations made for clinical samples do not translate into cause-effect relationship in vitro. Stimulation with exogenous ILs slightly improves colonic cell viability, accompanied by marginal upregulation of proliferation marker *Ki67* and anti-apoptotic *BCL2* in HCT 116 and HT-29 cells. It has a more marked effect on cell motility, accompanied by consistent upregulation of *CLDN2*, encoding tight junction protein claudin-2, and, significantly so in Caco-2 cells, by the downregulation of epithelial marker *TJP1*. Further research is needed to confirm the associations observed here on protein level and to elucidate involved signaling pathways.

## Figures and Tables

**Table 1:** Characteristics of study population for analysis of local interleukin expression at mRNA level.

Characteristics	ESCC	GC	CRC	*p*
*n*	16	14	21	-
Sex (F/M), *n*	6/10	3/11	7/14	0.619 ^1^
Age [yrs.], mean ± SD	63.3 ± 7	67.3 ± 10	67.7 ± 11.4	0.431 ^2^
Stage (I/II/III/IV)	0/5/10/1	3/3/5/3	5/9/7/0	0.064 ^1^
Primary tumor, T (1/2/3/4)	0/5/8/3	2/2/8/2	1/5/15/0	0.241 ^1^
Lymph node metastasis, N (no/yes)	8/8	6/8	14/7	0.261 ^1^
Distant metastasis, M (no/yes)	15/1	11/3	21/0	0.067 ^1^

*n*, number of observations; F/M, female-to-male ratio; yrs., years; SD, standard deviation; ^1^, Chi-squared test; ^2^, one-way ANOVA; ESCC, esophageal squamous cell carcinoma; GC, gastric adenocarcinoma; CRC, colorectal adenocarcinoma.

**Table 2:** Characteristics of study population for analysis of local interleukin expression at protein level.

Characteristics	ESCC	GC	CRC	*p*
*n*	18	14	17	-
Sex (F/M), *n*	8/10	3/11	7/10	0.365 ^1^
Age [yrs.], mean ± SD	64.1 ± 5.6	67.3 ± 10	66.8 ± 11.3	0.567 ^2^
Stage (I/II/III/IV)	2/6/9/1	3/3/5/3	4/8/5/0	0.248 ^1^
Primary tumor, T (1/2/3/4)	1/6/9/2	2/1/9/2	1/5/11/0	0.424 ^1^
Lymph node metastasis, N (no/yes)	9/9	6/8	12/5	0.261 ^1^
Distant metastasis, M (no/yes)	17/1	11/3	17/0	0.084 ^1^

*n*, number of observations; F/M, female-to-male ratio; yrs., years; SD, standard deviation; ^1^, Chi-squared test; ^2^, one-way ANOVA; ESCC, esophageal squamous cell carcinoma; GC, gastric adenocarcinoma; CRC, colorectal adenocarcinoma.

**Table 3:** Association of demographic and pathological data with IL-4 protein concentration in the GIT cancer patients.

Data	Fold-Change [Tumor-to-Adjacent] in IL-4 Protein Concentration		
	CRC	ESCC	GC
Age	r =0, *p* = 0.897 ^1^	r = 0, *p* = 0.930 ^1^	r = 0.46, *p* = 0.099 ^1^
Sex F vs. M	1.6 vs. 4.2, *p* = 0.199 ^2^	7.9 vs. 9.2, *p* = 0.844 ^2^	10.1 vs. 3.1, *p* = 0.392 ^3^
TNM	ρ = 0.11, *p* = 0.679 ^4^	ρ = 0.15, *p* = 0.553 ^4^	ρ = 0.53, *p* = 0.051 ^4^
T	ρ = 0.20, *p* = 0.452 ^4^	ρ = 0.08, *p* = 0.762 ^4^	ρ = 0.46, *p* = 0.100 ^4^
N0 vs. N ≥ 1	3.5 vs. 2.2, *p* = 0.568 ^2^	6.6 vs. 10.7, *p* = 0.528 ^2^	3.7 vs. 19.6, *p* = 0.349 ^2^
M0 vs. M1	-	-	2.1 vs. 17.6, *p* = 0.024 ^3^
G	ρ = 0.36, *p* = 0.161 ^4^	ρ = 0.73, *p* < 0.001 ^4^	ρ = 0.32, *p* = 0.271 ^4^

^1^ Data analyzed with Pearson correlation test (*r*); ^2^ analyzed with *t*-test for independent samples; ^3^ analyzed with Mann-Whitney U test; ^4^ analyzed with Spearman rank correlation (ρ). GIT, gastrointestinal tract; CRC, colorectal adenocarcinoma; ESCC, squamous cell carcinoma of esophagus; GC, gastric adenocarcinoma; F, females; M, males; TNM, tumor-node-metastasis grading system; T, depth of invasion (primary tumor extension); N, lymph node metastasis; M, distant metastasis; G, grade.

**Table 4:** Association of demographic and pathological data with *IL4* mRNA expression in the GIT cancer patients.

Data	Fold-Change [Tumor-to-Adjacent] in *IL4* Transcript Number		
	CRC	ESCC	GC
Age	r = 16, *p* = 0.475 ^1^	r = 21, *p* = 0.444 ^1^	r = −0.22, *p* = 0.440 ^1^
Sex F vs. M	1.7 vs. 0.6, *p* = 0.331 ^2^	12.2 vs. 1.4, *p* = 0.206 ^2^	0.3 vs. 1.2, *p* = 0.172 ^2^
TNM	ρ = −0.14, *p* = 0.548 ^3^	ρ= −0.21, *p* = 0.427 ^3^	ρ = 0.06, *p* = 0.834 ^3^
T	ρ = −0.37, *p* = 0.096 ^3^	ρ= −0.17, *p* = 0.529 ^3^	ρ = −0.06, *p* = 0.829 ^3^
N0 vs. N ≥ 1	0.7 vs. 1.3, *p* = 0.549 ^2^	17.4 vs. 0.6, *p* = 0.030 ^2^	0.6 vs. 1.2, *p* = 0.490 ^2^
M0 vs. M1	-	-	1.0 vs. 0.7, *p* = 0.747 ^2^
G	ρ = 0.01, *p* = 0.796 ^3^	ρ= 0.08, *p* = 0.759 ^3^	ρ = 0, *p* = 0.973 ^3^

^1^ Data analyzed with Pearson correlation test (*r*); ^2^ analyzed with *t*-test for independent samples; ^3^ analyzed with Spearman rank correlation (ρ). GIT, gastrointestinal tract; CRC, colorectal adenocarcinoma; ESCC, squamous cell carcinoma of esophagus; GC, gastric adenocarcinoma; F, females; M, males; TNM, tumor-node-metastasis grading system; T, depth of invasion (primary tumor extension); N, lymph node metastasis; M, distant metastasis; G, grade.

**Table 5:** Association of demographic and pathological data with *IL4Ra* mRNA expression in the GIT cancer patients.

Data	Fold-Change [Tumor-to-Adjacent] in *IL4Ra* Transcript Number		
	CRC	ESCC	GC
Age	r = 22, *p* = 0.347 ^1^	r = 25, *p* = 0.355 ^1^	r = 0, *p* = 0.999 ^1^
Sex F vs. M	1.1 vs. 0.7, *p* = 0.191 ^2^	1 vs. 1.6, *p* = 0.255 ^2^	4 vs. 2.9, *p* = 0.854 ^2^
TNM	ρ = −0.05, *p* = 0.815 ^3^	ρ = 0.06, *p* = 0.839 ^3^	ρ = −0.25, *p* = 0.383 ^3^
T	ρ = −0.07, *p* = 0.772 ^3^	ρ = 0.13, *p* = 0.627 ^3^	ρ = −0.25, *p* = 0.390 ^3^
N0 vs. N ≥ 1	0.9 vs. 0.8, *p* = 0.729 ^2^	1.9 vs. 1, *p* = 0.074 ^2^	3 vs. 3.2, *p* = 0.958 ^2^
M0 vs. M1	-	-	4.1 vs. 0.2, *p* = 0.492 ^2^
G	ρ = −0.04, *p* = 0.863 ^3^	ρ = 0.47, *p* = 0.077 ^3^	ρ = 0.05, *p* = 0.861 ^3^

^1^ Data analyzed with Pearson correlation test (*r*); ^2^ analyzed with *t*-test for independent samples; ^3^ analyzed with Spearman rank correlation (ρ). GIT, gastrointestinal tract; CRC, colorectal adenocarcinoma; ESCC, squamous cell carcinoma of esophagus; GC, gastric adenocarcinoma; F, females; M, males; TNM, tumor-node-metastasis grading system; T, depth of invasion (primary tumor extension); N, lymph node metastasis; M, distant metastasis; G, grade.

**Table 6:** Association of demographic and pathological data with IL-13 protein concentration in the GIT cancer patients.

Data	Fold-Change [Tumor-to-Adjacent] in IL-13 Protein Concentration		
	CRC	ESCC	GC
Age	r = 23, *p* = 0.366 ^1^	r = −0.19, *p* = 0.449 ^1^	r = 0.34, *p* = 0.231 ^1^
Sex F vs. M	1.4 vs. 1.5, *p* = 0.565 ^2^	2 vs. 2.2, *p* = 0.740 ^2^	2.2 vs. 1.4, *p* = 0.136 ^2^
TNM	ρ = 0.54, *p* = 0.024 ^3^	ρ = −0.02, *p* = 0.940 ^3^	ρ = −0.13, *p* = 0.658 ^3^
T	ρ = 0.30, *p* = 0.251 ^3^	ρ = 0.12, *p* = 0.631 ^3^	ρ = 0.14, *p* = 0.643 ^3^
N0 vs. N ≥ 1	1.3 vs. 1.9, *p* = 055 ^2^	2 vs. 2, *p* = 0.965 ^2^	2 vs. 1.4, *p* = 0.155 ^2^
M0 vs. M1	-	-	1.9 vs. 1.4, *p* = 0.506 ^2^
G	ρ = −0.07, *p* = 0.797 ^3^	ρ = −0.37, *p* = 0.137 ^3^	ρ = 0.15, *p* = 0.601 ^3^

^1^ Data analyzed with Pearson correlation test (*r*); ^2^ analyzed with *t*-test for independent samples; ^3^ analyzed with Spearman rank correlation (ρ). GIT, gastrointestinal tract; CRC, colorectal adenocarcinoma; ESCC, squamous cell carcinoma of esophagus; GC, gastric adenocarcinoma; F, females; M, males; TNM, tumor-node-metastasis grading system; T, depth of invasion (primary tumor extension); N, lymph node metastasis; M, distant metastasis; G, grade.

**Table 7:** Association of demographic and pathological data with *IL13* expression in the GIT cancer patients.

Data	Fold-Change [Tumor-to-Adjacent] in *IL13* Expression		
	CRC	ESCC	GC
Age	r = 25, *p* = 0.266 ^1^	r = −0.24, *p* = 0.367 ^1^	r = 0.1, *p* = 0.736 ^1^
Sex F vs. M	14.8 vs. 9, *p* = 0.736 ^2^	1.2 vs. 5.4, *p* = 0.483 ^2^	0.2 vs. 6.8, *p* = 0.145 ^2^
TNM	ρ = −0.56, *p* = 0.009 ^3^	ρ = -0.34, *p* = 0.192 ^3^	ρ = 0.13, *p* = 0.647 ^3^
T	ρ = −0.16, *p* = 0.490 ^3^	ρ = -0.24, *p* = 0.381 ^3^	ρ = −0.27, *p* = 0.344 ^3^
N0 vs. N ≥ 1	40.7 vs. 0.7, *p* = 002 ^2^	4.9 vs. 1.9, *p* = 0.650 ^2^	1.5 vs. 6, *p* = 0.485 ^2^
M0 vs. M1	-	-	2.2 vs. 13.5, *p* = 0.460 ^2^
G	ρ = −0.04, *p* = 0.863 ^3^	ρ = 0.01, *p* = 0.968 ^3^	ρ = −0.24, *p* = 0.417 ^3^

^1^ Data analyzed with Pearson correlation test (*r*); ^2^ analyzed with *t*-test for independent samples; ^3^ analyzed with Spearman rank correlation (ρ). GIT, gastrointestinal tract; CRC, colorectal adenocarcinoma; ESCC, squamous cell carcinoma of esophagus; GC, gastric adenocarcinoma; F, females; M, males; TNM, tumor-node-metastasis grading system; T, depth of invasion (primary tumor extension); N, lymph node metastasis; M, distant metastasis; G, grade.

**Table 8:** Association of demographic and pathological data with *IL13Ra1* expression in the GIT cancer patients.

Data	Fold-Change [Tumor-to-Adjacent] in *IL13Ra1* Expression		
	CRC	ESCC	GC
Age	r = 19, *p* = 0.406 ^1^	r = −0.21, *p* = 0.436 ^1^	r = −0.23, *p* = 0.434 ^1^
Sex F vs. M	0.8 vs. 1, *p* = 0.570 ^2^	0.9 vs. 1.1, *p* = 0.658 ^2^	1.1 vs. 1.3, *p* = 0.670 ^2^
TNM	ρ = 0.03, *p* = 0.910 ^3^	ρ = 0.05, *p* = 0.844 ^3^	ρ =−0.08, *p* = 0.774 ^3^
T	ρ = −0.08, *p* = 0.723 ^3^	ρ = 0.18, *p* = 0.517 ^3^	ρ = 0.14, *p* = 0.641 ^3^
N0 vs. N ≥ 1	0.9 vs. 1, *p* = 749 ^2^	1.4 vs. 0.7, *p* = 0.144 ^2^	1.1 vs. 1.4, *p* = 0.618 ^2^
M0 vs. M1	-	-	1.3 vs. 1, *p* = 0.547 ^2^
G	ρ = 0, *p* = 1 ^3^	ρ = 0.03, *p* = 0.903 ^3^	ρ = 0.20, *p* = 0.490 ^3^

^1^ Data analyzed with Pearson correlation test (*r*); ^2^ analyzed with *t*-test for independent samples; ^3^ analyzed with Spearman rank correlation (ρ). GIT, gastrointestinal tract; CRC, colorectal adenocarcinoma; ESCC, squamous cell carcinoma of esophagus; GC, gastric adenocarcinoma; F, females; M, males; TNM, tumor-node-metastasis grading system; T, depth of invasion (primary tumor extension); N, lymph node metastasis; M, distant metastasis; G, grade.

**Table 9:** Correlation pattern between fold-change in expression (tumor-to adjacent) of *IL4*, *IL4Ra*, *IL13*, and *IL13Ra1* and expression of representative mediators relevant for cancer growth in CRC patients.

Fold-Change in Expression	*IL13*	*IL13Ra1*	*IL4*	*IL4Ra*
*ACTA2*	ns	ns	0.39 ^4^	0.57 ^2^
*BCLxL*	ns	ns	ns	ns
*BCL2*	ns	ns	ns	ns
*CCL2*	ns	ns	ns	0.67 ^3^
*CDKN1A*	ns	0.60 ^2^	ns	0.48 ^1^
*CLDN2*	ns	ns	ns	−0.44 ^1^
*GLUT1*	ns	ns	ns	ns
*HIF1A*	ns	0.40 ^4^	ns	0.42 ^4^
*Ki67*	ns	ns	ns	−0.48 ^1^
*NOS2*	ns	ns	ns	ns
*ODC1*	ns	ns	ns	ns
*PTGS2*	ns	ns	ns	0.50 ^1^
*TJP1*	ns	ns	ns	0.68 ^3^
*VEGFA*	ns	0.53 ^1^	ns	0.48 ^1^

Data presented as Spearman correlation coefficients (ρ); ^1^, *p* < 0.05; ^2^, *p* < 0.01; ^3^, *p* < 0.001; ^4^, tendency (0.1 > *p* > 0.05); ns, not significant.

**Table 10:** Correlation pattern between expression of *IL4*, *IL4Ra*, *IL13*, and *IL13Ra1* and expression of representative mediators relevant for cancer growth in tumor and tumor-adjacent non-cancerous tissue from CRC patients.

Expression	Tumor Tissue				Adjacent non-Cancerous Tissue			
	*IL13*	*IL13Ra1*	*IL4*	*IL4Ra*	*IL13*	*IL13Ra1*	*IL4*	*IL4Ra*
*ACTA2*	ns	0.47 ^1^	ns	0.64 ^2^	ns	ns	ns	0.58 ^2^
*BCLxL*	ns	ns	ns	0.40 ^4^	ns	ns	ns	ns
*BCL2*	ns	0.43 ^4^	ns	0.57 ^2^	−0.39 ^4^	ns	ns	ns
*CCL2*	ns	0.51 ^2^	ns	0.56 ^2^	ns	ns	0.47 ^1^	ns
*CDKN1A*	ns	0.47 ^1^	ns	0.46 ^1^	ns	ns	ns	ns
*CLDN2*	ns	ns	ns	−0.39 ^4^	ns	ns	ns	ns
*GLUT1*	ns	ns	ns	ns	−0.41 ^4^	ns	ns	ns
*HIF1A*	ns	0.68 ^3^	ns	0.51 ^1^	ns	ns	0.41 ^4^	ns
*Ki67*	ns	ns	ns	ns	ns	0.42 ^4^	ns	−0.46 ^1^
*NOS2*	ns	ns	−0.42 ^4^	−0.42 ^4^	−0.41 ^4^	ns	ns	ns
*ODC1*	ns	ns	ns	ns	ns	ns	ns	ns
*PTGS2*	0.42 ^4^	ns	ns	ns	0.47 ^1^	ns	0.41 ^4^	0.64 ^2^
*TJP1*	ns	0.63 ^2^	ns	0.65 ^2^	ns	0.57 ^2^	ns	0.44 ^4^
*VEGFA*	0.39 ^4^	0.63 ^2^	ns	0.56 ^2^	ns	0.40 ^4^	ns	ns

Data presented as Spearman correlation coefficients (ρ); ^1^, *p* < 0.05; ^2^, *p* < 0.01; ^3^, *p* < 0.001; ^4^, tendency (0.1 > *p* > 0.05); ns, not significant.

**Table 11:** Characteristics of study population for analysis of systemic interleukin concentration.

Characteristics	CN	ESCC	CC	GC	CRC	*p*
*n*	36	93	32	31	71	-
Sex (F/M), *n*	16/20	32/61	7/25	10/21	27/44	0.375 ^1^
Age [yrs.], mean ± SD	65.4 ± 8.1	62.4 ± 8.5	62.3 ± 8.5	64.1 ± 9.7	65.8 ± 10.3	0.110 ^2^
Stage (I/II/III/IV/x)	-	9/21/25/38	1/2/4/26	1/5/5/20	7/28/29/6/1	<0.001 ^1^
Primary tumor, T (1/2/3/4/x)	-	11/17/24/41	0/1/3/28	1/2/9/19	1/8/39/22/1	<0.001 ^1^
Lymph node metastasis, N (no/yes/x)	-	34/59	2/30	6/25	35/35/1	<0.001 ^1^
Distant metastasis, M (no/yes/x)	-	55/38	6/26	11/20	64/6/1	<0.001 ^1^

*n*, number of observations; F/M, female-to-male ratio; yrs., years; SD, standard deviation; x, not determined; ^1^, Chi-squared test; ^2^, one-way ANOVA; CN, normal controls; ESCC, esophageal squamous cell carcinoma; CC, adenocarcinoma of the gastric cardia; GC, gastric adenocarcinoma; CRC, colorectal adenocarcinoma.

**Table 12:** Primers’ sequences.

Symbol	Gene Name	Accession No.	Primer Sequence 5′→3′	Amp. Size [bp]
*IL4 ^1^*	Interleukin 4	NM_000589.4	F: ccgtaacagacatctttgctgcc R: gagtgtccttctcatggtggct	108
*IL13 ^1^*	Interleukin 13	NM_002188.3	F: acggtcattgctctcacttgcc R: ctgtcaggttgatgctccatacc	160
*IL4Ra ^1^*	Interleukin 4 receptor alpha	NM_000418.4	F: ctgctcatggatgacgtggtca R: ggtgtgaactgtcaggtttcctg	138
*IL13Ra1 ^1^*	Interleukin 13 receptor subunit alpha 1	NM_001560.3	F: cctgaatgagaggatttgtctgc R: cagtcacagcagactcaggatc	125
*ACTA2 ^1^*	Alpha smooth muscle actin	NM_001141945.2	F: ctatgcctctggacgcacaact R: cagatccagacgcatgatggca	115
*BCL2 ^1^*	B-cell lymphoma 2	NM_000633.3	F: atcgccctgtggatgactgagt R: gccaggagaaatcaaacagaggc	127
*BCLxL ^1^*	B-cell lymphoma-extra large	NM_001317919.2	F: gccacttacctgaatgaccacc R: aaccagcggttgaagcgttcct	131
*CCL2 ^1^*	Monocyte chemoattractant protein 1 (MCP1)	NM_002982.4	F: agaatcaccagcagcaagtgtcc R: tcctgaacccacttctgcttgg	98
*CDKN1A ^1^*	Cyclin Dependent Kinase Inhibitor 1A (p21^CIP1/WAF1^)	NM_001220777.2	F: aggtggacctggagactctcag R: tcctcttggagaagatcagccg	95
*GAPDH ^2^*	Glyceraldehyde-3-phosphate dehydrogenase	NM_001256799.3	F: tagattattctctgatttggtcgtattgg R: gctcctggaagatggtgatgg	223
*CLDN2 ^1^*	Claudin 2	NM_020384.4	F: gtgacagcagttggcttctcca R: ggagattgcactggatgtcacc	153
*GLUT1 ^1^*	Glucose transporter 1	NM_006516.4	F: ttgcaggcttctccaactggac R: cagaaccaggagcacagtgaag	113
*HIF1A ^1^*	Hypoxia-inducible factor 1α	NM_181054.3	F: tatgagccagaagaacttttaggc R: cacctcttttggcaagcatcctg	145
*Ki67 ^1^*	Proliferation marker Ki67	NM_001145966.2	F: gaaagagtggcaacctgccttc R: gcaccaagttttactacatctgcc	151
*NOS2 ^1^*	Inducible nitric oxide synthase	NM_000625.4	F: gctctacacctccaatgtgacc R: ctgccgagatttgagcctcatg	136
*ODC1 ^1^*	Ornithine decarboxylase	NM_001287189.2	F: ccaaagcagtctgtcgtctcag R: cagagattgcctgcacgaaggt	162
*PTGS2 ^1^*	Prostaglandin-endoperoxide synthase 2 (COX2)	NM_000963.4	F: cggtgaaactctggctagacag R: gcaaaccgtagatgctcaggga	156
*TJP1 ^1^*	Tight junction protein 1	NM_001355014.2	F: gtccagaatctcggaaaagtgcc R: ctttcagcgcaccataccaacc	132
*VEGFA ^1^*	Vascular endothelial growth factor A	NM_001025366.3	F: ttgccttgctgctctacctcca R: gatggcagtagctgcgctgata	126

Amp., amplicon; ^1^, primer sequences were as proposed by Origene (www.origene.com); ^2^, primers were designed using Beacon Designer Probe/Primer Design Software (BioRad), validated in silico by Blast analysis, and their specificity tested by means of melting curve analysis and an electrophoresis in a high-resolution agarose. Forward and reverse primer sequences are denoted by “F” and “R”, respectively.

## Abbreviations

CC	Adenocarcinoma of gastric cardia
CRC	Colorectal adenocarcinoma
ESCC FBS	Esophageal squamous cell carcinoma Fetal bovine serum
GC	Gastric adenocarcinoma
GIT	Gastrointestinal tract
IL	Interleukin
EMT	Epithelial-mesenchymal transition
NRQSRB	Normalized relative quantitiesSulforhodamine B
TNM	Tumor-node-metastasis grading system
